# Long non-coding RNA *HIF1A-As2* and MYC form a double-positive feedback loop to promote cell proliferation and metastasis in KRAS-driven non-small cell lung cancer

**DOI:** 10.1038/s41418-023-01160-x

**Published:** 2023-04-11

**Authors:** Kaixin Yang, Wenyang Zhang, Linghui Zhong, Yinan Xiao, Sudhakar Sahoo, Matteo Fassan, Kang Zeng, Peter Magee, Michela Garofalo, Lei Shi

**Affiliations:** 1 RNA Oncology Group, School of Public Health, Lanzhou University, 730000 Lanzhou, People’s Republic of China; 2 Computational Biology Support, Cancer Research UK Manchester Institute, University of Manchester, Alderley Park, Manchester, SK10 4TG UK; 3 Department of Medicine, Surgical Pathology & Cytopathology Unit, University of Padua, Padua, 35100 Italy; 4 Imaging & Cytometry Facility, Cancer Research UK Manchester Institute, University of Manchester, Alderley Park, Manchester, SK10 4TG UK; 5 Transcriptional Networks in Lung Cancer Group, Cancer Research UK Manchester Institute, University of Manchester, Alderley Park, Manchester, SK10 4TG UK

**Keywords:** Metastasis, Genetics research

## Abstract

Lung cancer is the leading cause of cancer-related deaths worldwide. *KRAS* is the main oncogenic driver in lung cancer that can be activated by gene mutation or amplification, but whether long non-coding RNAs (lncRNAs) regulate its activation remains unknown. Through gain and loss of function approaches, we identified that lncRNA *HIF1A-As2*, a KRAS-induced lncRNA, is required for cell proliferation, epithelial-mesenchymal transition (EMT) and tumor propagation in non-small cell lung cancer (NSCLC) in vitro and in vivo. Integrative analysis of *HIF1A-As2* transcriptomic profiling reveals that *HIF1A-As2* modulates gene expression in trans, particularly regulating transcriptional factor genes including MYC. Mechanistically, *HIF1A-As2* epigenetically activates MYC by recruiting DHX9 on MYC promoter, consequently stimulating the transcription of MYC and its target genes. In addition, KRAS promotes *HIF1A-As2* expression via the induction of MYC, suggesting *HIF1A-As2* and MYC form a double-regulatory loop to strengthen cell proliferation and tumor metastasis in lung cancer. Inhibition of *HIF1A-As2* by LNA GapmeR antisense oligonucleotides (ASO) significantly improves sensitization to 10058-F4 (a MYC-specific inhibitor) and cisplatin treatment in PDX and KRAS^LSLG12D^-driven lung tumors, respectively.

## Introduction

Lung cancer is the most common cause of cancer-related death worldwide with more than 1.5 million deaths annually [1, 2]. NSCLC accounts for a large proportion of lung cancer patients, and its 5-year survival rate ranges from 10 to 15% depending on stages and regional variations [3]. Due to the complex mutational landscape, clinical therapeutic strategies against NSCLC have been proved largely ineffective. A consistent portion of the molecular alterations responsible for NSCLC initiation and progression arise from the *KRAS* oncogene. *KRAS* belongs to a family of small GTPases, which switches between an “on” and an “off” conformation by guanine nucleotide exchange factors (GEFs) and GTPase activating proteins (GAPs) [4, 5]. *KRAS* mutations frequently occur in exon 2 at codon 12 and these alterations give rise to lung cancer development. After many efforts in the past decades to develop a direct inhibitor for *KRAS*, drugs that specifically inhibit KRAS G12C mutations are beginning to be available in the clinic [6, 7]. Because the specific inhibitor for KRAS G12D is still in the pre-clinical stage, identifying essential downstream targets in KRAS G12D-driven tumor is critical.

LncRNAs are a class of non-coding transcripts longer than 200 nucleotides (nt) in length, exclusively expressed in specific tissues and involved in numerous physiological and pathological processes [8]. LncRNAs have been well-established as a prominent layer of association with multiple macromolecules, for example, DNA, chromatin, proteins and other RNA transcripts that regulate oncogenic pathways thus contributing to the proliferation, viability and motility of cancer cells, making them appealing as novel anti-cancer therapeutic targets [9, 10]. Studies have reported that several lncRNAs, such as *HOTAIR* and *MetaLnc9*, are implicated in lung cancer tumorigenesis [11, 12], suggesting that regulation of these lncRNAs is an attractive tool to investigate cancer progression and drug resistance.

We recently showed that lncRNA *KIMAT1* modulates KRAS signalling, microRNA biogenesis and tumor metastasis through interactions with the RNA-binding proteins DHX9 and NPM1 in lung cancer [13]. Here, we discovered that *HIF1A-As2*, a KRAS-responsive lncRNA, is upregulated in lung cancer and its high expression is associated with a poor patient prognosis. *HIF1A-As2* is required for cell proliferation, EMT and tumor metastasis. *HIF1A-As2* guides its binding protein DHX9 to stimulate MYC signalling, whereas activated MYC, in turn, could transcriptionally activate *HIF1A-As2* expression, suggesting *HIF1A-As2* and MYC form a double-positive loop that may robustly alter downstream genes and tumor growth. Small molecules targeting *HIF1A-As2* and MYC significantly inhibit tumor growth, suggesting lncRNA *HIF1A-As2* presents an important biological and clinical impact in lung cancer, particularly in KRAS-driven NSCLC.

## Results

### *HIF1A-As2* is a KRAS-responsive lncRNA

**Fig. 1:**
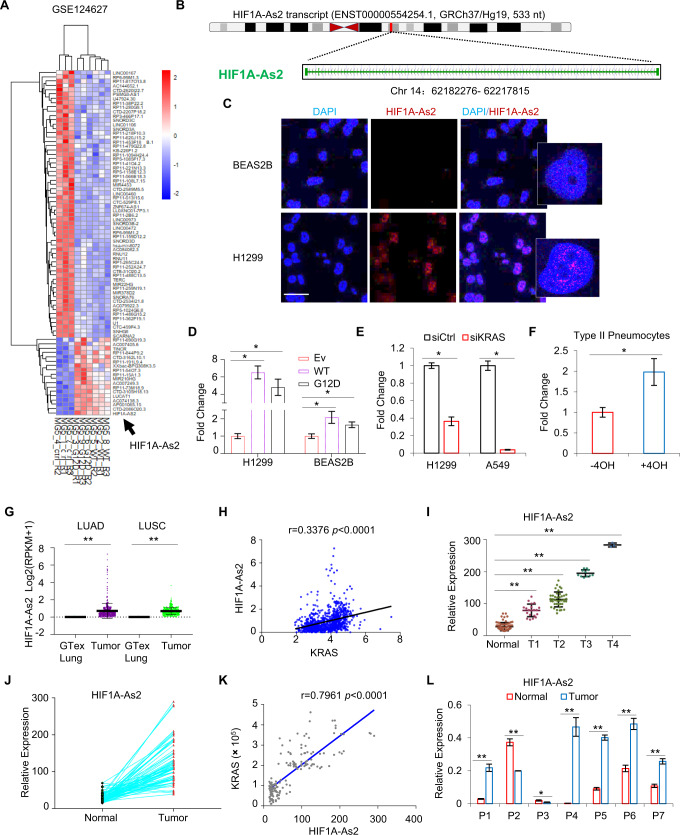
*HIF1A-As2* is upregulated in NSCLC. **A** Heatmap showing the differentially expressed lncRNAs upon overexpression of KRAS WT or KRAS G12D in H1299 cells. **B** Schematic annotation of *HIF1A-As2* transcript on chromosome 14. **C** Representative images of smFISH indicating the localization of *HIF1A-As2* in BEAS2B and H1299 cells. DAPI, blue; *HIF1A-As2*, red. Scale bar, 75 μm. **D** RT-qPCR showing increased expression of *HIF1A-As2* in H1299 and BEAS2B cells transfected with KRAS WT or KRAS G12D compared to control cells. **E** RT-qPCR showing downregulation of *HIF1A-As2* after silencing of KRAS in H1299 and A549 cells. **F** Endogenous *HIF1A-As2* level in Type II Pneumocytes cells treated with 4-Hydroxytamoxifen (4OH) that induces KRAS G12V. **G** *HIF1A-As2* level in the TCGA LUAD (*n* = 540) and LUSC (*n* = 501) dataset compared to GTex Lung cohorts (*n* = 427). **H** Positive correlation between KRAS and *HIF1A-As2* (LUAD + GTex *n* = 967). **I**,**J** *HIF1A-As2* is highly expressed in late-stage vs early-stage (**I**) and in tumor vs normal samples (**J**) in Biomax LUAD paired TMA samples (Biomax LUAD normal *n* = 75, T1 *n* = 20, T2 *n* = 40, T3 *n* = 12, T4 *n* = 3). **K** Positive correlation between *HIF1A-As2* and KRAS in samples from the Biomax LUAD paired TMAs, normal *n* = 75, tumor *n* = 75. **L** RT-qPCR showing increased expression of *HIF1A-As2* in KRAS mutant tumor (*n* = 7) vs normal samples (*n* = 7) from the MCRC Biobank LUAD TMAs. **D**–**F**, **L** Data show mean ± S.D (*n* = 3). **p value < 0.001, *p value < 0.05 by two-tailed Student’s *t* test.

We previously carried out a transcriptomic profiling analysis in H1299 cells with ectopic expression of KRAS WT or KRAS G12D (GSE124627) and identified 104 unique differentially expressed lncRNAs following KRAS stimulation, whereas *HIF1A-As2* was the topmost induced lncRNA (Fig. 1A, Supplementary Fig 1A). *HIF1A-As2* is a 533 nt lncRNA that resides on chromosome 14 and is one of the two antisense transcripts of *HIF1A*, a master transcriptional activator that regulates many hypoxia-induced oncogenes [14]. *HIF1A-As2* contains three exons with only one isoform (Fig. 1B), lacks coding potential and is not conserved across species (Supplementary Fig 1B, C). The full length and sequence of the *HIF1A-As2* transcript were determined by the 5′ and 3′ rapid-amplification of cDNA ends (RACE) assay (Supplementary Fig. 1D). RNA single molecular in situ hybridization (smFISH) and cell fractionation following subcellular RT-qPCR revealed that *HIF1A-As2* is predominately localized in the nucleus (Fig. 1C, Supplementary Fig 1E), suggesting that *HIF1A-As2* might play a role in transcriptional regulation or chromatin interaction. *HIF1A-As2* is associated with cell survival, migration and invasion in diverse cancers [15–17]. However, the functions of *HIF1A-As2* in lung cancer, particularly in KRAS-driven NSCLC, are unknown. Therefore, we aimed to investigate the underlying function of *HIF1A-As2* in NSCLC. First, we used RT-qPCR to validate the regulation of *HIF1A-As2* by KRAS WT or KRAS G12D in multiple cells, including H1299, A549, BEAS2B and Type II pneumocytes, an inducible KRAS^G12V^ immortalized cell line [18]. Results showed that KRAS overexpression increased, whereas KRAS silencing decreased *HIF1A-As2* expression (Fig. 1D–F). In addition, EGFR silencing (KRAS upstream) with siRNA or ERKs inhibition (KRAS downstream) with Trametinib led to *HIF1A-As2* downregulation in H1299 and 549 cells (Supplementary Fig 1F, G). Furthermore, KRAS and *HIF1A-As2* were highly expressed in the lung adenocarcinoma (LUAD) and lung squamous cell carcinoma (LUSC) compared to the normal lung from TCGA datasets (Fig. 1G, Supplementary Fig 1H), elevated in late-stage compared to early-stage tumors from a second independent cohort of Biomax LUAD paired TMA samples (Fig. 1I, J; Supplementary Fig 1J). In addition, we observed a positive correlation existed between KRAS and *HIF1A-As2* in TCGA and TMA samples (Fig. 1H, K, Supplementary Fig 1I). RT-qPCR analysis also detected higher expression of *HIF1A-As2* in five of seven KRAS mutant lung cancers compared to matched normal lungs from MCRC Biobank LUAD TMAs samples (Fig. 1L). These results suggest that *HIF1A-As2* is a KRAS-responsive lncRNA that may contribute to the development of KRAS-driven NSCLC.

### *HIF1A-As2* affects cell survival and proliferation in vitro

**Fig. 2:**
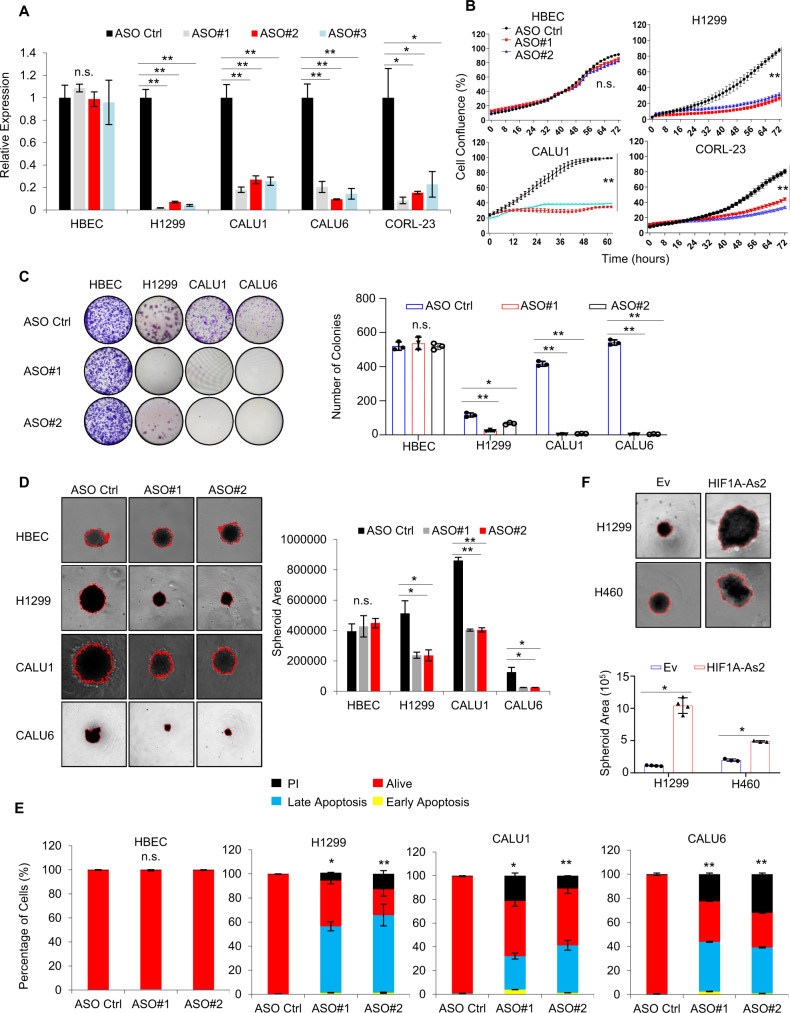
*HIF1A-As2* modulates cell proliferation in vitro. **A** RT-qPCR showing silencing of *HIF1A-As2* in HBEC, H1299, CALU1, CALU6 and CORL-23 cells upon three different ASOs. **B** IncuCyte assay showing inhibition of cell growth after silencing of *HIF1A-As2* by two different ASOs. **C** and **D** *HIF1A-As2* silencing inhibits cell colony formation (**C**) and 3D spheroid formation (**D**) in multiple NSCLC cell lines. **E** Quantification of Annexin-V apoptosis assay after knocking down of *HIF1A-As2* with ASOs in multiple cell lines. **F** Stably expressing *HIF1A-As2* promotes 3D spheroid formation in H1299 and H460 cells. Data show mean ± S.D (**A**–**E**, **F** H460 cell line, *n* = 3; **F** H1299 cell line, *n* = 4). **p value < 0.001, *p value < 0.05 by two-tailed Student’s *t* test.

Gene Set Enrichment Analysis (GSEA) showed the enrichment of the G2M checkpoint signature in KRAS WT and G12D gene sets (Supplementary Fig. 2A). In line with the fact that KRAS influences cell proliferation in various cancers [19], we hypothesized that *HIF1A-As2* may participate in lung tumorigenesis. Firstly, we examined the expression of *HIF1A-As2* in multiple NSCLC cell lines. *HIF1A-As2* was highly expressed in CORL23, CALU1, CALU6 and H1299 cells, which present more mesenchymal phenotypes, compared to other NSCLC cell lines (Supplementary Fig. 2B). We silenced *HIF1A-As2* and performed functional studies to assess its functions in NSCLC. We designed three different ASOs, which trigger degradation of the lncRNA-ASO duplex by RNase H [20], and observed a substantial downregulation of *HIF1A-As2* by RT-qPCR in H1299, CALU1, CALU6 and CORL23 cells with high expression of *HIF1A-As2*, but not in the HBEC cells which have no or low expression of *HIF1A-As2* (Fig. 2A, Supplementary Fig. 2B). The precise silencing of *HIF1A-As2* was validated by smFISH to guarantee the specificity of the ASO and prevent off-targets (Supplementary Fig. 2C). *HIF1A-As2* silencing dramatically reduced cell proliferation and clonogenic ability in several lung cancer cells carrying KRAS amplification or mutation, but not in normal lung cell lines (Fig. 2B–D, Supplementary Fig. 2D). Furthermore, *HIF1A-As2* silencing with ASO or specific siRNA facilitates H1299 and CALU6 cell sensitization to gefitinib and cisplatin treatment, respectively (supplementary Fig. 2E, F). Silencing of *HIF1A-As2* in H1299 cells also promoted a significant arrest in G1 and G2 phases (Supplementary Fig. 2G). In addition, we observed prominently increased cell apoptosis upon *HIF1A-As2* KD with ASOs in diverse NSCLC cell lines but not in normal cells (Fig. 2E, Supplementary Fig. 3).

Consistently, cells that stably expressing *HIF1A-As2* exhibited a significant increase in colony proliferation and 3D spheroid formation (Fig. 2F, Supplementary Fig. 4A, B), suggesting that *HIF1A-As2* acts as an oncogene in NSCLC. In addition, the overexpression of *HIF1A-As2* overturned the phenotype by *HIF1A-As2* deficiency, highlighting the specific influence of *HIF1A-As2* ASO in NSCLC (Supplementary Fig. 4C).

### *HIF1A-As2* modulates EMT by sponging microRNA-200c

Emerging studies have reported that lncRNAs play crucial regulatory roles in tumorigenesis by acting as competing endogenous RNAs (ceRNAs), which can sponge microRNAs (miRNAs) and interfere with miRNA-mediated degradation of target mRNAs in cancer [21]. We next investigated whether *HIF1A-As2* reacts to cell processes by antagonizing microRNAs. *HIF1A-As2* had been shown to be a target of microRNA-200c by two different algorithms (Supplementary Fig. 5A). We next utilized the LncTar database to predict the potential binding regions and performed a reporter assay (Supplementary Fig. 5B) [22]. The dual-luciferase assay indicated that ectopic miR-200c repressed the reporter activity of pGL3-*HIF1A-As2*, whereas this phenomenon was rescued after mutating the binding sites (Supplementary Fig. 5C). Next, we performed a pulldown assay and observed miR-200c is enriched in the *HIF1A-As2* pulldown lysate, revealing miR-200c interacts with *HIF1A-As2* (Supplementary Fig. 5D). Furthermore, RT-qPCR showed that overexpression of miR-200c inhibited *HIF1A-As2*, with a positive control of ZEB1 (Supplementary Fig. 5E). These results indicate that miR-200c directly targets and inhibits *HIF1A-As2*. As miR-200c is a well-established EMT regulator [23], we wondered if *HIF1A-As2* is involved in this process. Interestingly, gene set enrichment analysis revealed that EMT signature was significantly enriched in the *HIF1A-As2* KD gene set (Supplementary Fig. 5F). *HIF1A-As2* knock down inhibited wound closure (Supplementary Fig. 5G, H), cell migration and invasion (Supplementary Fig. 5I), and mesenchymal marker TFAP4 and SNAIL expression (Supplementary Fig. 5J). Cells stably expressing *HIF1A-As2* displayed an elongated phenotype compared to parental cells (Supplementary Fig. 5K). In summary, these data conclude that *HIF1A-As2* modulates EMT as a ceRNA of microRNA-200c.

### *HIF1A-As2* promotes tumor growth and metastasis in vivo

**Fig. 3:**
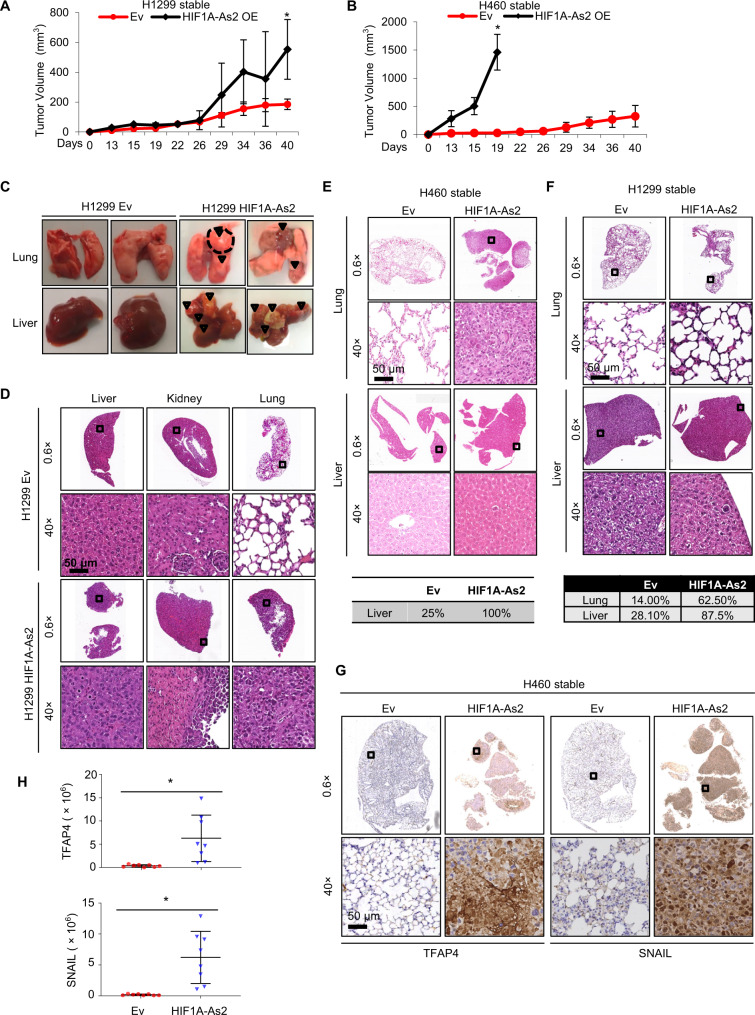
*HIF1A-As2* promotes tumor growth and metastasis in vivo. **A** and **B** Tumor growth curves of xenograft mice injected with H1299 (**A**) and H460 (**B**) cell lines that stably expressing *HIF1A-As2* compared to control mice. Data show mean ± S.D (**A**, *n* = 7; **B**, *n* = 8). **C** Representative images of lungs and livers 5 weeks after percutaneously injected into the left lateral thorax of NSG mice with H1299 cells stably expressing *HIF1A-As2* or empty vector. **D** Representative H&E staining of livers, kidneys and lungs from mice orthotopically injected with H1299-*HIF1A-As2* or controls cells. Scale bar, 50 μm. **C**,**D** *n* = 7 per group. **E** Top: Representative H&E staining of lungs and livers from NSG mice with orthotopic injection of H460-*HIF1A-As2* or control cells into the left lateral thorax. Scale bar, 50 μm. Bottom: Table showing the percentages of tissues with metastasis in the indicated groups. *n* = 8 per group. **F** Top: Representative H&E staining of lungs and livers from NSG mice with injection of H1299 cells in the tail vein. Bottom: Table showing the percentages of tissues with metastasis in the indicated groups. Ev *n* = 7, H1299-*HIF1A-As2* *n* = 8. **G** and **H** IHC images and quantification of TFAP4 and SNAIL from the mice with orthotopic injection of H460-*HIF1A-As2* or control cells. **p value < 0.001, *p value < 0.05 by two-tailed Student’s *t* test.

Next, we examined the effect of *HIF1A-As2* on tumor growth via different mouse models. Murine tumor xenografts derived from H1299 and H460 cells stably expressing *HIF1A-As2* displayed a remarkable growth advantage compared to control tumors (Fig. 3A, B, Supplementary Fig. 6A). Next, we orthotopically injected the stably expressing *HIF1A-As2* cells into the lungs of NOD/SCID Gamma (NSG) mice to assess the capacity of tumor initiation and metastasis. Overexpression of *HIF1A-As2* promoted tumor development, malignant ascites and distant metastases in the liver and kidney (Fig. 3C–E, Supplementary Fig. 6B, C). We also injected H1299 stably expressing *HIF1A-As2* cells into the tail vein of NSG mice and found increased metastases in the lung and liver compared to mice in the control group (Fig. 3F). In addition, we observed the significant upregulation of mesenchymal marker TFAP4 and SNAIL in the mice with orthotopic injection of H460 *HIF1A-As2* cells compared to the control cells (Fig. 3G, H). Altogether, we concluded that *HIF1A-As2* is associated with cell proliferation in vitro and in vivo.

### *HIF1A-As2* directly interacts with DHX9

**Fig. 4:**
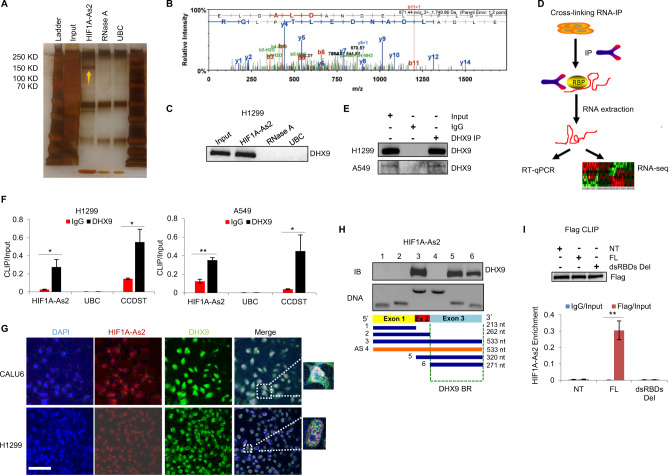
*HIF1A-As2* directly interacts with DHX9. **A** Silver staining of cell lysates upon *HIF1A-As2* pull-down with biotinylated antisense probes after UV crosslinking in H1299 cells. Samples pre-treated with RNase A or pull-down with housekeeping gene *UBC* were used as negative controls. The yellow arrow indicates the band for DHX9. **B** The *HIF1A-As2* pull-down cell lysate were performed with Mass Spectrometry. Mass Spectrometry profiling showing the peptide sequences of DHX9 listed on the top of the graph. Data are representative of two independent biological replicates. **C** Immunoblotting showing the DHX9 interacts with *HIF1A-As2*. **D** Schematic representation of Cross-linking RNA Immunoprecipitation (CLIP) assay. **E** Immunoblotting confirming the immunoprecipitation of DHX9. **F** CLIP assay indicating the enrichment of *HIF1A-As2* in the DHX9 antibody-bound complex. *UBC* and lncRNA *CCDST* were used as negative and positive controls, respectively. **G** Sequential immunofluorescence (IF) and smFISH representative images showing the co-localization of *HIF1A-As2* and DHX9 in CALU6 and H1299 cells. DAPI, blue; *HIF1A-As2*, red; DHX9, green. Scale bar, 75 μm. **H** Construct mapping of the binding region of *HIF1A-As2* to DHX9. Bottom, Diagrams of full-length and different truncated *HIF1A-As2* constructs. Middle, In vitro transcribed *HIF1A-As2* constructs with according sizes. Top, Immunoblotting analysis for DHX9 upon pull-down by different *HIF1A-As2* truncated constructs in H1299 cells. **I** CLIP assay showing the direct interaction between *HIF1A-As2* and DHX9. Flag-tagged DHX9 FL, DHX9 dsRBDs Del or empty vector (NT) was co-transfected with pCDH-*HIF1A-As2* plasmid in BEAS2B cells for 48 h. CLIP was performed with Flag-specific antibody (Top) and *HIF1A-As2* level was examined by RT-qPCR (Bottom). Data show mean ± S.D (*n* = 3). **p value < 0.001, *p value < 0.05 by two-tailed Student’s *t* test.

LncRNAs exert their functions by interacting with RNA-binding proteins (RBPs) [24]. Therefore, we purified endogenous *HIF1A-As2* RNA complexes using biotinylated RNA antisense probes to analyze the potential RBPs by RNA antisense purification coupled with mass spectrometry (RAP-MS) [25, 26]. A sample pre-digested with RNase A or incubated with the *Ubiquitin C* (*UBC*) probes was used as negative controls (Fig. 4A). Seven proteins were reproducibly identified in two biological replicates (Supplementary Table 1) and DHX9 attracted our attention for its role in cancer progression (Fig. 4B) [27]. DHX9 is a highly conserved DExD/H-box protein, expressed in the nucleus and the cytoplasm, involved in many processes including transcriptional activation, RNA editing, microRNA biogenesis and tumor cell maintenance [28]. Silver staining and immunoblotting assays showed a clear band for DHX9 in the sample pulled down with the *HIF1A-As2* probes, while no signal was observed after purification with the *UBC* probe or in the lysate pre-treated with RNase A (Fig. 4A and C). We further performed a Cross-linking RNA immunoprecipitation (CLIP) assay to confirm the interaction between lncRNA and protein (Fig. 4D). *HIF1A-As2* was significantly enriched in the DHX9 antibody-bound complexes compared to the immunoglobulin G (IgG)-bound sample. *UBC* and lncRNA *CCDST* were used as negative and positive controls, respectively (Fig. 4E, F) [29]. In accordance, sequential immunofluorescence and smFISH showed co-localization of *HIF1A-As2* and DHX9 in the nucleus (Fig. 4G), suggesting a possible interaction. Next, we performed an in vitro RNA pulldown assay with *HIF1A-As2* full-length or truncated fragments to map the *HIF1A-As2* functional motifs that bind to DHX9. A region of 271 nt at the 3′ end of *HIF1A-As2* was identified as an essential DHX9-binding region (Fig. 4H). To confirm the direct interaction, we further performed the CLIP assay using a construct that overexpressed DHX9 full-length (FL), a truncated DHX9 construct lacking the double-strand RNA-binding domains (dsRBDs Del), or an empty vector in the normal lung cells BEAS2B, which has low expression of *HIF1A-As2*. We observed that *HIF1A-As2* was exclusively enriched in the lysate from DHX9 FL but not in DHX9 dsRBD Del, implying that *HIF1A-As2* directly binds to DHX9 and the dsRBDs of DHX9 are essential for the interaction (Fig. 4I).

Following that, we investigated the modulation between *HIF1A-As2* and DHX9. Interestingly, *HIF1A-As2* OE or silencing did not alter DHX9 levels in H1299 cells, however, DHX9 could improve the RNA stability of *HIF1A-As2* in both H1299 and CALU6 cells (Supplementary Fig. 7A, B). In addition, *HIF1A-As2* positively correlated with DHX9 in LUAD samples (Supplementary Fig. 7C), and high expression of *HIF1A-As2* and DHX9 was associated with a worse prognosis in LUAD (Supplementary Fig. 7D). We cloned *HIF1A-As2* full-length (*HIF1A-As2* FL) and a truncated construct (*HIF1A-As2* Δ2) in a pCDH lentiviral vector and performed a colony formation assay to determine whether the deletion of the DHX9-binding site in *HIF1A-As2* could offset the biological effects mediated by this lncRNA. Overexpression of the truncated construct gave rise to a lower number of colonies compared to cells transfected with *HIF1A-As2* FL constructs (Supplementary Fig. 7E). In addition, DHX9 KO hampered the promotion of cell growth by ectopic *HIF1A-As2*, indicating that the interaction with DHX9 is important for *HIF1A-As2*-promoted tumor growth (Supplementary Fig. 7F). DHX9 full length but not the DHX9 dsRBDs del which missing the *HIF1A-As2* binding domain could reverse the inhibited cell growth by DHX9 KO (Supplementary Fig. 7G), suggesting the important roles of dsRBD domain in lung cancer. In vivo, H1299 DHX9 KO cells were orthotopically injected into the lungs of NSG mice. Fewer distant metastases were observed in DHX9 KO mice compared to control mice (Supplementary Fig. 7H–J). Altogether, these data show that DHX9 is a direct binding protein of *HIF1A-As2* and the oncogenic roles of *HIF1A-As2* are, in part, mediated by DHX9.

### *HIF1A-As2* recruits DHX9 on MYC promoter and activates MYC expression

**Fig. 5:**
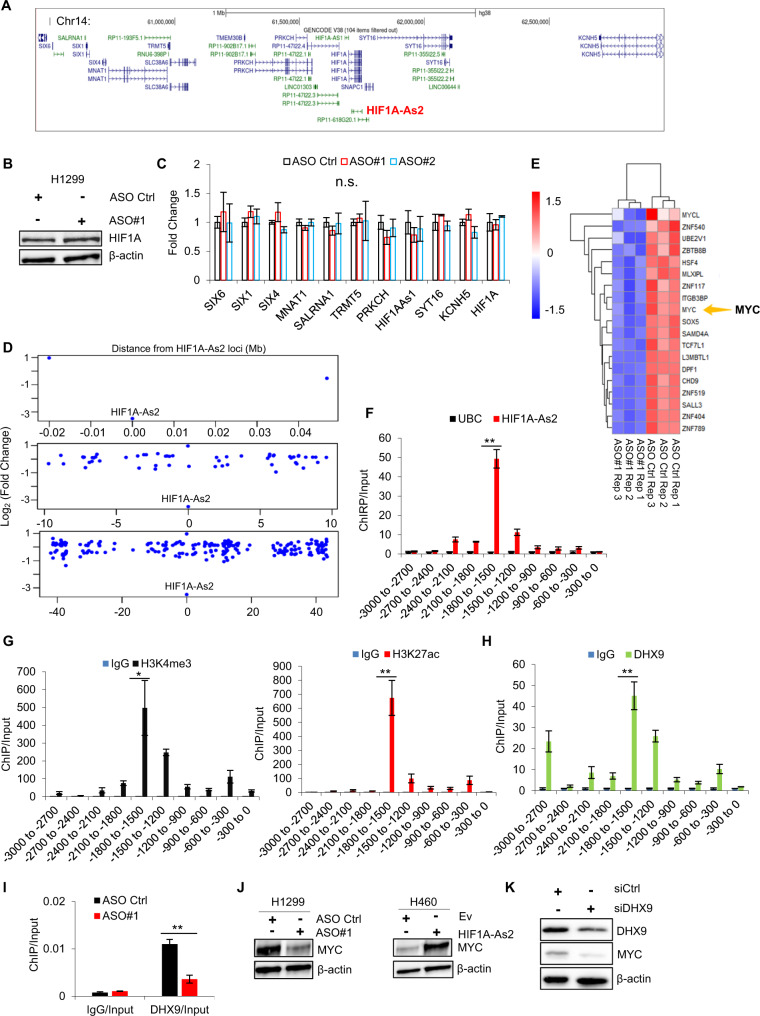
*HIF1A-As2* recruits DHX9 onto MYC promoter to trigger MYC expression. **A** UCSC Genome Browser displaying the neighboring genes (within 1 Mb distance) of *HIF1A-As2*. **B** and **C** Immunoblotting (**B**) and RT-qPCR (**C**) showing *HIF1A-As2* does not regulate nearby genes. **D** Scatterplot depiction of the differential expression of genes from RNA-seq in the genomic vicinity of *HIF1A-As2*. Differentially expression was calculated as log_2_(Fold Change) of *HIF1A-As2* KD/Ctrl. **E**, Heatmap of representative TF expression level from transcriptomic profiling after silencing of *HIF1A-As2*. **F** ChIRP-qPCR analysis of *HIF1A-As2* enrichment on MYC promoter region. *HIF1A-As2* bound to the MYC promoter region (−1800 to −1500 nt). **G** and **H** ChIP-qPCR showing enrichment of H3K4me3, H3K27ac (**G**) and DHX9 (**H**) on MYC promoter region. **I** ChIP-qPCR showing the less enrichment of DHX9 on MYC promoter upon *HIF1A-As2* disruption. **J** Silencing of *HIF1A-As2* inhibits, while stable expression of *HIF1A-As2* promotes MYC expression in H1299 and H460 cells, respectively. **K** Immunoblotting showing downregulation of MYC upon DHX9 silencing in H1299 cells. Data show mean ± S.D (*n* = 3). **p value < 0.001, *p value < 0.05 by two-tailed Student’s *t* test.

LncRNAs can modulate genes in trans or *in cis* [30]. In our study, we observed that the depletion of *HIF1A-As2* with two different ASOs did not alter the expression level of neighboring genes (Fig. 5A–C), suggesting that it may regulate genes in trans. We performed a genome transcriptome analysis to better examine the genes modulated by *HIF1A-As2* and observed a total number of 2217 differentially expressed genes upon *HIF1A-As2* KD (GSE124628). Furthermore, leveraging the transcriptome microarray data revealed that genes regulated by *HIF1A-As2* depletion were predominantly located more than 1 Mb away (Fig. 5D). GSEA analysis showed that *HIF1A-As2* regulated important pathways including nucleic acid binding, regulation of transcription and chromatin DNA binding (Supplementary Fig. 8A), in line with the localization of *HIF1A-As2* in the nucleus, this suggests *HIF1A-As2* may regulate genes at the transcriptional or post-transcriptional level. Of note, the most downregulated genes among the differentially expressed genes were those transcriptional factors (TFs) in the deficiency of *HIF1A-As2* with two different ASOs (Fig. 5E, Supplementary Fig. 8B).

It is interesting to note that one of the most dysregulated TFs by *HIF1A-As2* KD is MYC, an important oncogene that modulates cell proliferation, cell cycle and apoptosis in cancer [31]. GSEA analysis confirmed that MYC signaling pathway was one of the enriched gene signatures in *HIF1A-As2* KD gene sets (Supplementary Fig. 8C, D). In addition, the venny plot showed that *HIF1A-As2*-regulated genes overlapped with MYC-targeted genes obtained from the Harmonizome dataset (Supplementary Fig. 8E, Left) [32]. We also knocked down DHX9 in H1299 cells and conducted a transcriptome microarray (GSE124626). Interestingly, a total number of 208 genes were communally regulated by *HIF1A-As2* and DHX9, suggesting a functional regulation between *HIF1A-As2* and DHX9 (Supplementary Fig. 8F). Notably, the genes regulated by DHX9 KD also significantly overlapped with MYC targets (Supplementary Fig. 8E, Right). These results indicate that the prominent oncogenic functions of *HIF1A-As2* are, at least in part, due to MYC regulation and its downstream pathway. We, therefore, focused on the MYC gene.

We first performed a chromatin isolation by RNA purification (ChIRP) assay that pulled down *HIF1A-As2* with biotinylated labelled antisense probes, followed by qPCR analysis to examine if *HIF1A-As2* binds to the promoter region of MYC [33]. We observed that the *HIF1A-As2* deposited on a specific region of −1800 to −1500 nt upstream of the transcription start sites (TSSs) of the MYC promoter (Fig. 5F), overlapping with enrichment of the activating histone markers H3K4me3 and H3K27ac (Fig. 5G), which suggests this fragment was the binding loci for *HIF1A-As2*. In addition, we performed a ChIP assay and found that *HIF1A-As2* bound to H3K4me3 and H3K27ac (Supplementary Fig. 8G).

Given the fact that DHX9 transcriptionally regulates genes, we further sought whether *HIF1A-As2* affects the occupancy of DHX9 on MYC promoter loci. The ChIP experiment pointed out that DHX9 is also bound to the −1800 to −1500 nt region of MYC promoter (Fig. 5H). Substantially, *HIF1A-As2* KD or stably overexpression resulted in less or enhanced enrichment of DHX9 on MYC promoter (Fig. 5I, Supplementary Fig. 8H). In addition, immunoblotting results demonstrated that MYC was downregulated upon *HIF1A-As2* and DHX9 silencing, while upregulated by *HIF1A-As2* OE (Fig. 5J and K). In conclusion, *HIF1A-As2* coordinates with DHX9 to foster MYC expression.

### *HIF1A-As2*/DHX9 axis promotes cell proliferation through MYC target genes

**Fig. 6:**
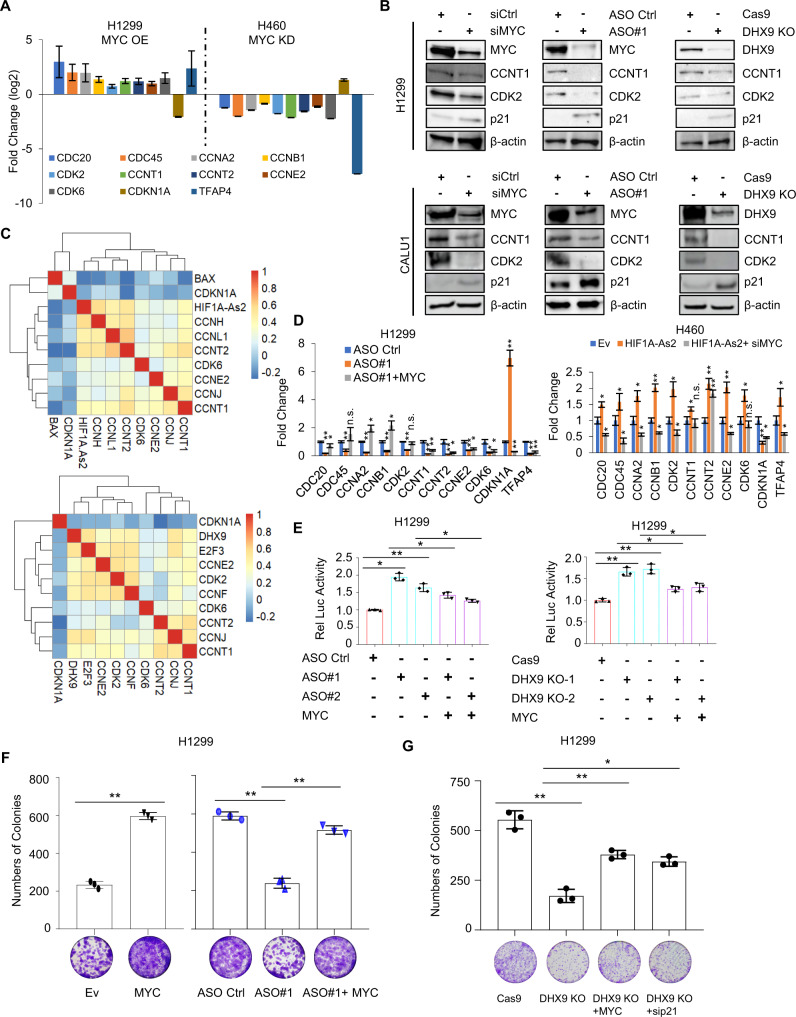
*HIF1A-As2* promotes cell proliferation via MYC target genes. **A** Expression of the indicated MYC targets upon MYC OE or KD in H1299 and H460, respectively. **B** Immunoblotting analysis of the indicated proteins upon MYC KD, *HIF1A-As2* KD and DHX9 KO in H1299 and CALU1. **C** Correlation analysis between *HIF1A-As2* and MYC targets or DHX9 and MYC targets in TCGA LUAD dataset (*n* = 540). **D** Administration of MYC reversed the regulation of the MYC targets by *HIF1A-As2* in H1299 and H460. **E** Reporter assay showing restored MYC attenuated the increased luciferase activities of p21 reporter upon *HIF1A-As2* KD or DHX9 KO. Rel Luc, relative luciferase. **F** Colony assay showing the MYC hampered the *HIF1A-As2*-regulated colony formation in H1299 cells. **G** DHX9 regulates cell growth via MYC and p21 in H1299 cells. Data show mean ± S.D (*n* = 3). **p value < 0.001, *p value < 0.05 by two-tailed Student’s *t* test.

MYC drives cell growth via a variety of target genes including *CDC20*, *CCNA2*, *CCNB1*, *CCNE2*, *CDK6* and *CDKN1A*, which are important for cell proliferation and apoptosis [31, 34–38]. We next explored if these MYC target genes are affected by *HIF1A-As2*. First, the ENCODE dataset and ChIP-qPCR assay showed that MYC is directly bound to the promoter loci of these genes in NSCLC (Supplementary Fig. 9). In addition, qPCR showed the upregulation of *CDC20*, *CDC45*, *CCNA2*, *CCNB1*, *CDK2*, *CCNT1*, *CCNT2*, *CCNE2*, *CDK6* and *TFAP4*, and the downregulation of *CDKN1A* upon MYC OE in H1299 cells whereas vice versa in H460 cells (Fig. 6A). We also observed similar results upon *HIF1A-As2* KD or DHX9 KO (Fig. 6B, D) and a significant correlation between *HIF1A-As2* and MYC targets or between DHX9 and MYC targets in LUAD (Fig. 6C). However, the dysregulation of target genes by *HIF1A-As2* was rescued with the re-enforced expression or silencing of MYC, respectively (Fig. 6D). In addition, we discovered that upregulated MYC and lower p21 were found in orthotopic mice with *HIF1A-As2* overexpression (Supplementary Fig. 10A, B), whereas MYC was downregulated and p21 was elevated in orthotopic mice with DHX9 KO (Supplementary Fig. 10C).

We next performed a luciferase activity assay with the reporter of a target gene (e.g., the p21-Luc promoter) and proved that knock down of MYC with siRNA-pools or 10058-F4, a specific MYC inhibitor, increased the luciferase activities of the p21 promoter, whereas restored MYC can prevent this downregulation (Supplementary 11A). *HIF1A-As2*/DHX9 disruption or overexpression also altered the p21 reporter activities. However, these phenomena were reversed by regained or silenced MYC (Fig. 6E, Supplementary Fig. 11B), indicating that *HIF1A-As2*/DHX9 indirectly regulates the MYC targets.

Based on these findings, we next sought to determine whether *HIF1A-As2*/DHX9 regulate cell growth or apoptosis via the MYC targets. Colony formation assay showed that MYC overexpression impaired colony formation by *HIF1A-As2* KD in H1299 and CALU1 (Fig. 6F, Supplementary 11C). MYC KD could antagonize *HIF1A-As2*-promoted colony formation in H1299 and H460 (Supplementary 11D, E). Similar outcomes were observed in DHX9 KO experiments (Fig. 6G). In addition, overexpression of MYC altered the inhibited 3D spheroid formation by *HIF1A-As* KD or DHX9 KO, respectively (Supplementary Fig. 12). Moreover, the Annexin-V assay showed that MYC OE or p21 KD impaired the induction of apoptosis by two distinct *HIF1A-As2* ASOs in H1299 and CALU1 (Supplementary 13). In summary, these data demonstrate that *HIF1A-As2* and DHX9 regulate cell behavior via MYC target genes in NSCLC.

### KRAS activates *HIF1A-As2* via induction of MYC

**Fig. 7:**
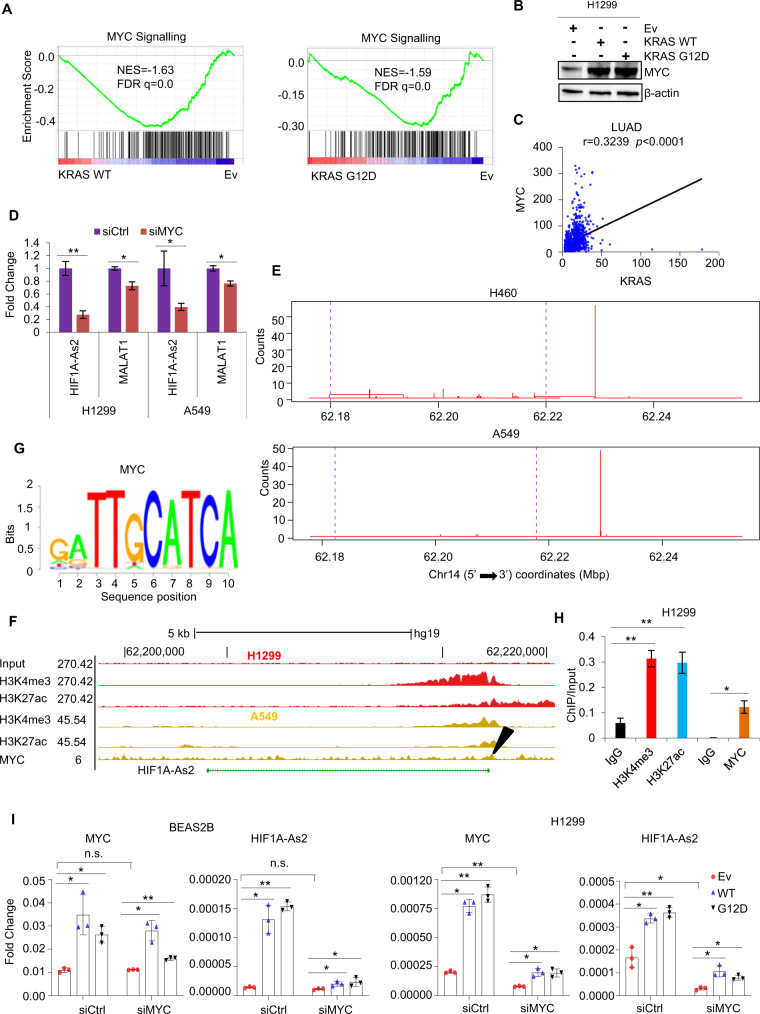
KRAS activates *HIF1A-As2* through MYC. **A** MYC gene signature is enriched in KRAS WT or KRAS G12D gene sets. **B** Immunoblotting showing upregulated MYC by overexpressed KRAS WT or KRAS G12D. **C** KRAS is significantly correlated with MYC in LUAD corhorts (GTex + LUAD = 965). **D** RT-qPCR for *HIF1A-As2* level after silencing of MYC in H1299 and A549 cells. *MALAT1* was used as a positive control. **E** CAGE-seq revealing the potential transcription start sites of *HIF1A-As2*. CAGE-seq counts were defined by FANTOM5 mammalian promoter expression atlas in H460 and A549 cells. Purple dashed lines show the start position and end position of *HIF1A-As2*. **F** UCSC Genome Browser showing the ChIP-seq signals of H3K4me3, H3K27ac and MYC on the *HIF1A-As2* promoter locus. **G** MEME analysis for the binding motifs of transcription factor MYC on *HIF1A-As2* promoter locus. **H** ChIP-qPCR showing the enrichment of MYC on *HIF1A-As2* promoter in H1299 cells. H3K4me3 and H3K27ac were used as positive controls. **I** The induction of *HIF1A-As2* by KRAS WT or KRAS G12D was abolished by MYC KD in BEAS2B (Left) and H1299 (Right) cells. **D**, **H** and **I** Data show mean ± S.D (*n* = 3). **p value < 0.001, *p value < 0.05 by two-tailed Student’s *t* test.

Next, we investigated the mechanism by which KRAS induces *HIF1A-As2* in NSCLC. We noted that the MYC gene signature was enriched in the KRAS WT and G12D gene sets (Fig. 7A). Ectopic KRAS WT and G12D increased MYC levels in H1299 (Fig. 7B) and KRAS positively correlated with MYC in LUAD (Fig. 7C). Consistently, MYC KD reduced *HIF1A-As2* expression in H1299 and A549 cells with *MALAT1* as a positive control (Fig. 7D) [39]. This observation suggests that MYC, a downstream gene of KRAS, may participate in the induction of *HIF1A-As2* by KRAS.

We next performed the Cap Analysis of Gene Expression sequencing (CAGE-seq) in lung cancer cell lines obtained from the FANTOM5 study to identify the 5’ Transcription Starting Site (TSS) on the *HIF1A-As2* promoter (Fig. 7E). A ChIP-seq experiment with histone markers H3K4me3 and H3K27ac (GSE124630) was applied to confirm the promoter region of *HIF1A-As2* (Fig. 7F). Interestingly, the H3K4me3 and H3K27ac signals overlapped with the MYC binding signal on the *HIF1A-As2* promoter, indicating MYC may control *HIF1A-As2* expression. We then used the online tool The MEME Suite [40] to characterize the binding motifs of MYC (Fig. 7G), and validated it by ChIP-qPCR in H1299 cells. Results showed that the *HIF1A-As2* promoter region was significantly enriched in MYC antibody-bound complexes compared to IgG. H3K4me3 and H3K27ac were used as positive controls (Fig. 7H). In addition, the induction of *HIF1A-As2* by KRAS WT or KRAS G12D was prevented by silencing of MYC in two different cell lines (Fig. 7I). In summary, these results confirm that KRAS induces *HIF1A-As2* through the transcriptional factor MYC.

### Targeting *HIF1A-As2* opens a therapeutic window for KRAS-driven NSCLC

**Fig. 8:**
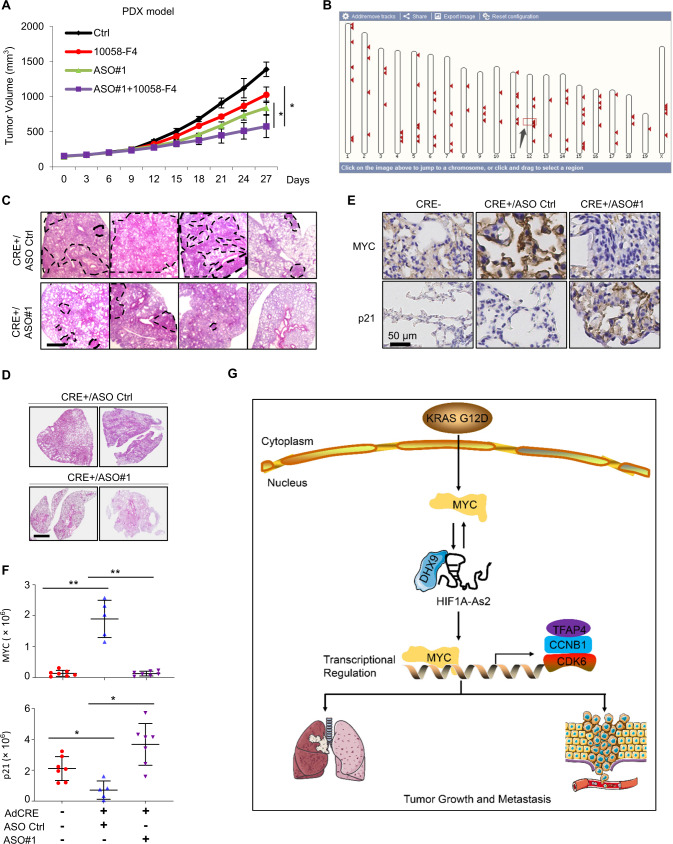
*HIF1A-As2* ASO inhibits tumorigenesis in vivo. **A** Tumor growing curves of PDX mice by intravenous inject with Ctrl, 10058-F4, ASO#1 and ASO#1 + 10058-F4. *n* = 4 per group. **B** Ensembl BLAST tool showing the pairwise sequence alignment of the human *HIF1A-As2* against the mouse genome. Black arrow shows the location of the most likely candidate gene on the mouse chromosome. **C** Representative images of high-power magnifications of lungs harvested from KRAS^LSLG12D^ mice treated with CRE +/ASO Ctrl or CRE +/ASO#1. Scale bar, 1 mm. Lung parenchyma affected by several epithelial lesions is highlighted by the dashed line. **D** Representative images of the whole lung sections from the two groups of mice as indicated in (**C**). Scale bar, 2 mm. **E** and **F** Representative images (**E**) and quantification (**F**) of IHC staining for MYC and p21 in the indicated groups from KRAS^LSLG12D^ mice treated with CRE−, CRE +/ASO Ctrl or CRE +/ASO#1. **C**–**F** Group GRE- *n* = 7, Group CRE +/ASO Ctrl *n* = 5, Group CRE +/ASO#1 *n* = 7. **G** Proposed model demonstrating the role and underlying mechanism of KRAS/*HIF1A-As2*/DHX9/MYC circuit in NSCLC pathogenesis and metastasis. Data show mean ± S.D. **p value < 0.001, *p value < 0.05 by two-tailed Student’s *t* test.

In the end, we investigated the potential implication of *HIF1A-As2* in KRAS-driven lung cancer. We first utilized a PDX model with transplantation of KRAS WT amplification lung tumor in NSG mice to determine the influence of alone or concomitant inhibition of *HIF1A-As2* and MYC on tumor growth. 10058-F4 treatment reduced MYC and *HIF1A-As2* expression in tumor (Supplementary Fig. 14A). *HIF1A-As2* KD by ASO significant repressed *MYC*, and mesenchymal markers *TFAP4* and *SNAIL* in tumor (Supplementary Fig. 14B). Synergistically silencing of *HIF1A-As2* enhances sensitization to 10058-F4 treatment in PDX tumor (Fig. 8A).

We next checked whether *HIF1A-As2* KD could abolish KRAS G12D-driven lung tumorigenesis. The public database indicates that *HIF1A-As2* is not conserved between human and mouse genomes (Supplementary Fig. 1C). Nevertheless, we used the Ensembl database to align the *HIF1A-As2* sequence against the mouse genome and discovered a transcript named *Gm15283* (ENSMUSG00000087700) localized on chr12:73,926,481-73,949,785 hitting 83% overlapping similarity (Fig. 8B, Black Arrow). Interestingly, *Gm15283* also is the antisense transcript of mouse *HIF1A* but does not code for any proteins (Supplementary Fig. 14C), indicating *Gm15283* is the equivalent gene of *HIF1A-As2* (Here, we refer to it as mouse *HIF1A-As2* for convenience). We employed mice carrying a conditional allele encoding KRAS G12D (*KRAS*^LSLG12D^ mouse model) to further define the therapeutic potential of ASO targeting *HIF1A-As2*. AdenoCRE inhalation, which activates KRAS G12D, caused an increase in *HIF1A-As2* expression 8 weeks after treatment (Supplementary Fig. 14D). Since we have shown that *HIF1A-As2* KD overrides drug resistance in vitro (Supplementary Fig. 2E, F), we next tested whether *HIF1A-As2* KD could affect lung tumorigenesis. We randomly divided mice into three groups: one group did not receive the AdenoCRE (AdCRE−), while the other two groups received the AdenoCRE and then were treated for 8 weeks (once a week) with either ASO control (CRE +/ASO Ctrl) or *HIF1A-As2* ASO (CRE +/ASO#1) plus two intraperitoneal doses of cisplatin. In comparison to CRE +/ASO Ctrl mice, mice that received CRE +/ASO#1 had significantly less neoplastic area as assessed by H&E analysis (Fig. 8C, D, Supplementary Fig. 14E). We also noticed increased weights of lungs, *HIF1A-As2*/Ki67/KRAS expression in CRE +/ASO Ctrl mice as compared to CRE- and CRE +/ASO#1 mice (Supplementary Fig. 14F–H). Furthermore, we discovered that MYC was downregulated and p21 was elevated in *KRAS*^LSLG12D^ mice with *HIF1A-As2* KD (Fig. 8E, F). Thus, these results strongly indicate that *HIF1A-As2* contributes to KRAS-driven lung tumor progression in vivo.

## Discussion

LncRNAs have emerged as novel master regulators of initiation and progression in a wide variety of tumors. In the present study, we identified that *HIF1A-As2* and MYC form a double-regulatory loop that enhances cell survival, tumor growth and metastasis, suggesting targeting *HIF1A-As2* could be a vulnerability in KRAS-dependent NSCLC (Fig. 8G).

Previous studies have demonstrated that *HIF1A-As2* promotes cell proliferation and migration in a variety of cancers [16, 41–43]. *HIF1A-As2* is also upregulated in lung cancer and is associated with poor outcomes [44, 45]. Intriguingly, they were mainly interested in the functions of *HIF1A-As2* by sponging microRNAs rather than other underlying mechanisms. In line with that, we also showed that *HIF1A-As2* is responsible for EMT processes by sequestering microRNA-200c. Besides, we further identified that *HIF1A-As2* enhances tumorigenesis via its interaction with DHX9. DHX9 is a key member of the DExD/H-box family of helicases which are involved in transcriptional regulation by working as coactivators or corepressors through the association with lncRNAs [28, 29, 46]. These divergent functions might be attributable to differences in cell type, tumor heterogeneity, or other unknown factors, suggesting the complexity of DHX9 in carcinogenesis. A previous study showed that *HIF1A-As2* modulates glioblastoma stem-like cell proliferation, self-renewal and hypoxia-dependent molecular reprogramming by interacting with DHX9 and further influencing downstream target HMGA1. Interestingly, they also demonstrated that silencing of *HIF1A-As2* does not regulate HIF1A and DHX9, which is consistent with our results, however the mechanism of how *HIF1A-As2*/DHX9 modulates downstream genes in glioblastoma was not included [17].

Many antisense lncRNAs can interfere directly with corresponding sense protein-coding genes (*cis*-regulatory) [8]. In contrast, lncRNAs can give rise to another distinctive feature in the form of *trans*-regulatory elements, which control genes at different chromosomes or homologous loci from where they are transcribed, as a consequence, regulating chromatin states, influencing nuclear structure and organization [30]. We revealed that *HIF1A-As2* deficiency does not regulate the host gene *HIF1A* and nearby genes, suggesting *HIF1A-As2* modulates gene expression in trans. We further found that *HIF1A-As2* can guide DHX9 to the promoter region of the transcriptional factor MYC, thereby fostering MYC signalling. MYC is the first described gene that encodes for an oncogenic transcription factor with significant importance for cell proliferation through multiple targets including CDKs, cyclins and cycle inhibitors such as CDKN1A (encoding p21 protein) [34, 47, 48]. Administration of MYC and p21 counteracts the influence of *HIF1A-As2* on cell proliferation, implying *HIF1A-As2* modulates cell proliferation via MYC signalling. In addition, we observed that KRAS promotes *HIF1A-As2* via the induction of MYC, suggesting *HIF1A-As2* and MYC can form a bidirectional regulation in NSCLC. In line with MYC is a well-known oncogenic factor in cancer, our findings provide a new target particularly in MYC-related tumor.

Modern advances technologies in the novel generation have gain a lot of attempts to develop the RNA-based therapeutics in a wide spectrum of applications [49]. In general, there are two approaches to developing RNA-based molecules: (1) RNA interference by short antisense oligonucleotides (ASOs) and messenger RNA (mRNA), where mRNAs encoding certain peptides or proteins elicit their transient expression in the cytoplasm. ASOs are short, synthetic and single-stranded oligodeoxynucleotides, that are complementary to target sequences, that can silence target gene levels by inducing of RNase H endonuclease activity and cleaves the RNA-DNA heteroduplex. Several ASOs have already been approved for human diseases including cancer [50, 51]. For example, lncRNA *HAND2-AS1* ASO dramatically inhibits HCC tumorigenesis [52]. LncRNA *MALAT1* KD by ASO represses breast cancer cell growth in vitro and in vivo [53]. Antisense oligonucleotides against *lncGATA6* exhibit strong therapeutic efficacy in colorectal cancer [54]. Here, we found that *HIF1A-As2* KD by ASO synergistically with the MYC-specific inhibitor significantly suppresses tumor growth in the PDX model. In addition, *HIF1A-As2* KD upon ASO significantly inhibits KRAS G12D-induced tumorigenesis. Given the broad adverse prognostic impact of KRAS mutation in NSCLC, targeting of *HIF1A-As2* and/or MYC by effective small-molecule compound inhibitors or agents holds potent therapeutic potential in treating KRAS-driven NSCLC.

There are a few limitations in this study. Firstly, we aligned the *HIF1A-As2* sequences in the mouse genome and identified a transcript called *Gm15283*, a potential counterpart of *HIF1A-As2* in the mouse. It is localized at the antisense of mouse HIF1A and is not a protein-coding gene. Due to a lack of mouse cell lines, we did not carry out the functional investigation in the present study.

In conclusion, our data depicts the existence of a double-positive regulatory loop that stabilizes the oncogenic axis of *HIF1A-As2*/DHX9/MYC and recapitulates the important signal transduction pathways. Once established, this self-regulatory circuit may sufficiently drive the absence of extrinsic or intrinsic signals and thus significantly affecting the important cell-dependent machinery. In summary, small-molecule mediated ablation of *HIF1A-As2* function would provide a therapeutic strategy for KRAS-driven NSCLC.

## Methods

### Cell lines

LUAD cell lines H1299, H460, A549, H1975, CALU1 and CALU6, lung fibroblasts HEL299, lung bronchial epithelial cell line HBEC, normal human bronchial epithelium cell line BEAS2B, kidney embryonic cells HEK293 were purchased from American Type Culture Collection (ATCC) and cultured as suggested by ATCC’s guidelines. CORL-23 cells line was purchased from Sigma-Aldrich. Type II pneumocytes cells were a kind gift from Professor Dr Julian Downward (The Institute of Cancer Research, London). All cell lines were validated by STR profiling and checked for Mycoplasma through in-house testing at the CRUK MI Molecular Biology Core Facility.

### Human tissue samples

Lung adenocarcinoma tissue microarrays (TMAs) (Biomax LUAD HLugA150CS02, normal *n* = 75, T1 *n* = 20, T2 *n* = 40, T3 *n* = 12, T4 *n* = 3) were purchased from US Biomax. TMAs containing lung adenocarcinoma samples with mutant *KRAS* and matched normal lungs were obtained from the Manchester Cancer Research Centre (MCRC) Biobank under the Christie Hospital Human Tissue License number 18/NW/0092 (MCRC Biobank normal *n* = 7, tumor *n* = 7). smFISH or IHC were used to examine the expression of *HIF1A-As2* and KRAS in tumor and matched normal lung samples, respectively. Images were acquired with a gSTED microscope and spots were counted using the online JAVA software of StarSearch for smFISH [55] and QuPath for IHC [56].

### Cell fractionation

Total nuclear and cytoplasmic extracts were obtained from cells cultured in 60 mm TC-treated culture dishes using the Cytoplasmic and Nuclear RNA Purification Kit (Norgen), according to the manufacturer’s instructions.

### LncRNA cloning and lentiviral transduction

Full-length and truncated constructs of *HIF1A-As2* were PCR amplified and inserted into a lentiviral vector (pCDH-GFP, System Biosciences). The inserted sequences were examined by sequencing. To produce lentiviruses with *HIF1A-As2* stable expression, HEK293 cells were transfected with pCDH-*HIF1A-As2* plasmid and package plasmids (Gag:pol, VSVG and REV) using Lipofectamine 2000 regent. Infectious lentiviruses were collected at 48, 72 and 96 h after transfection and filtered through 0.45 μm filters. H1299 cells were infected with the virus in the presence of polybrene (Santa Cruz) for 48 h and were sorted based on GFP expression using Flow Cytometry (Novocyte). Primers are listed in Supplementary Table 2.

### Dual-luciferase reporter assay

The full transcript of *HIF1A-As2* was PCR amplified into a pGL3 vector (Promega). 200 ng of pGL3 vector, 20 ng of Renilla plasmid (Promega), and 50 nM of oligos (Applied Biosystems) were co-transfected in H1299 cells for 48 h. A dual-Luciferase Assay (Promega) was used to examine luciferase activity. The deletion of binding sites was performed using the QuickChange Mutagenesis Kit (Stratagene). Primers are listed in Supplementary Table 2.

### RNA extraction and quantitative real-time PCR (RT-qPCR)

Total RNA was extracted from cells or tissues using the TRIzol method according to the manufacturer’s instructions. cDNA was synthesized from 1 μg of total RNA using the Verso cDNA Synthesis Kit (ThermoFisher Scientific), and real-time PCR was performed using SYBR Green PCR master mix (Applied Biosystems^TM^). Relative gene expression was calculated using −ΔΔCT normalized to *β-actin*. RNA integrity was verified on an Agilent Bioanalyzer 2100 (Agilent Technologies, Palo Alto, CA). Primer sequences are listed in Supplementary Table 3.

### Protein extraction and immunoblotting

Total protein lysates were homogenized in 1 × RIPA buffer (Sigma-Aldrich) plus protease inhibitors (Roche) and centrifuged at 13000 *rpm* for 20 min at 4 °C. The supernatant was used for immunoblotting with the indicated antibodies. Signal was detected using the Western Bright ECL-Spray substrate and a ChemiDoc instrument (Bio-Rad). Antibodies are listed in Supplementary Table 4.

### RNA stability assay

H1299 and CALU6 cells were grown into six-well plates. Actinomycin D (5 μg/mL, Sigma-Aldrich) was added to cells and cultured for the indicated time. Cells were collected at constant times for RNA extraction. The remaining *HIF1A-As2* was analyzed by RT-qPCR. *β-actin* was used for normalization.

### *HIF1A-As2* transcript boundaries and chromatin immunoprecipitation (ChIP)

*HIF1A-As2* transcription starting site (TSS) was identified using CAGE-seq counts data obtained from the FANTOM5 study (https://fantom.gsc.riken.jp/5/) for LUAD cell lines [57]. JASPAR was used to predict the likelihood of transcription factor binding sites in the *HIF1A-As2* sequence. ChIP assay was performed as previously described [58]. Online databases Encode and UCSC Genome Browser were used to visualize the H3K4me3, H3K27ac and MYC ChIP-seq signals on the *HIF1A-As2* promoter. Primers and antibodies are listed in Supplementary Table 3 and 4.

### siRNA, LNA GapmeR ASO and plasmid transfection

Commercially available ON-TARGET plus smart pool siRNAs for EGFR and MYC were purchased from Dharmacon. siRNAs for DHX9, KRAS and p21 were purchased from Thermofisher Scientific. Three different LNA GapmeR ASOs for *HIF1A-As2* were designed and synthesized from Qiagen. ASO (50 nM) or siRNA (50 nM) were transfected using Hiperfect reagent (Qiagen) for 48 h according to the manufacturer’s instructions. Plasmids were transiently transfected into the cells with Lipofectamine 2000 reagent (ThermoFisher Scientific). siRNA, ASO sequences and plasmid information are listed in Supplementary Table 5 and 6.

### Transcriptome analysis

RNA-seq reads were quality checked with FastQC

(https://www.bioinformatics.babraham.ac.uk/projects/fastqc/) and aligned in paired-end mode to the human genome assembly (GRCh37) using the RSubread package aligner with the default settings [59]. Mapped data were converted to gene-level integer read counts using feature Counts (RSubread package) and the Ensemble GTF annotation (Homo sapiens. GRCh 37.74). The expression of a gene was measured in RPKM (Reads Per Kilobase Million) units.

### Differentially expressed genes (DEGs) analysis

DEGs were analyzed by comparing the gene-level integer read count data for the overexpression or knock down and control samples using the DESeq2 Bioconductor package with default settings [60]. The resulting *p* values were adjusted (padj) using the Benjamini and Hochberg approach for controlling the false discovery rate (FDR). Genes with an adjusted *p* value determined to be <0.05 (FDR < 0.05) by DESeq2 with a fold change value ≥1.5 or ≤0.8 between the two groups were considered as differentially expressed.

### Overall survival analysis

Disease-free survival and overall survival (OS) analyses were performed on LUAD datasets. Kaplan–Meier curves were generated at 20 years follow-up period using samples for which both survival and expression data. BAM files for all these datasets were downloaded from GDC (https://portal.gdc.cancer.gov) and counts were extracted from the BAM files using feature Counts.

### Gene set enrichment analysis (GSEA)

Gene signature analysis was based on the MSigDB v6.2 database using the GSEA desktop implementation software [61]. Genes in the dataset were pre-ranked and weighted by the independent gene-level Wald statistics and 1000 phenotype-based permutations were conducted. GSEA was performed using the Hallmark and C6 oncogenic signatures to understand the functions of KRAS-, *HIF1A-As2*- or DHX9-regulated gene signatures. FDR values <0.05 were considered significant.

### Cross-linking RNA immunoprecipitation (CLIP)

CLIP was performed as previously described [62]. Cells were UV irradiated at 0.8 J/cm^2^, lysed in RIPA buffer (Sigma-Aldrich) with 1 × protease inhibitor cocktail (Sigma-Aldrich) and RNase inhibitor (NEB) for 10 min in ice. Lysates were then precleared with Protein G beads (Thermofisher Scientific) for 1 h at 4 °C, and immunoprecipitated with IgG or the indicated antibodies for 3 h at 4 °C. Immuno-complexes were precipitated with Protein G beads and washed six times with washing buffer (50 mM Tris-HCl pH 7.5, 150 mM NaCl, 1 mM MgCl_2_, and 0.05% IGEPAL CA-630). 10% of beads were boiled with 1 × Laemli buffer (Bio-Rad) at 95 °C for 5 min and loaded on a polyacrylamide gel to verify immunoprecipitation efficiency. The remaining beads were treated with TurboDNase (Thermofisher Scientific) and Proteinase K (NEB). RNA was isolated using the TRIzol solution. Proper negative and/or positive control were applied in this experiment.

### Native RNA pull-down assay

The native RNA pull-down assay for *HIF1A-As2* was performed as previously described [13]. Primers are listed in Supplementary Table 2.

### Co-immunoprecipitation (Co-IP)

Co-IP assay was performed as previously described [13]. Antibodies are listed in Supplementary Table 4.

### MTS cell viability and IncuCyte cell growth analysis

5.0 × 10^3^ cells were cultured in 96-well plates. Cell viability was assessed using the CellTiter 96 Aqueous One Solution Cell Viability assay (Promega) and measured at 490 nm in a Multilabel Counter (SpectraMax M5).

Cell confluence was analyzed using the IncuCyte Zoom live-cell imaging system from Essen Bioscience. Pre-treated cells were seeded into a 96-well plate and phase-contrast images were taken every 2 h for a total of 72 h and the percentage of confluence was calculated with the Incucyte Zoom software.

### Cell cycle analysis and annexin-V assay

For cell cycle analysis, H1299 cells were transfected with ASOs for 48 h. After that, cells were washed with PBS and harvested with trypsin. Cell pellets were fixed with ice-cold 70% ethanol for 1 h and incubated overnight at −20 °C. Cell pellets were stained with propidium iodide solution (ThermoFisher Scientific) at 37 °C for 1 h. DNA content was analyzed using Flow Cytometry (NovoCyte).

For the Annexin-V assay, cells were grown in 6-well plates, transfected with ASOs for 48 h then washed with PBS and harvested with trypsin. Cell pellets were incubated with Annexin-V for 15 min (Trevigen) in the dark at room temperature. 400 μl 1 × binding buffer was added to the cells and the percentage of apoptotic cells was analyzed using Flow Cytometry (NovoCyte).

### Colony formation assay

5.0 × 10^3^ cells were seeded in each well of six-well plates. 14 days later, cells were subsequently washed with PBS and fixed with cold methanol, stained with 0.05% crystal violet (Sigma-Aldrich), photographed and counted using the GelCount System (Oxford Optronix) and the GelCount^TM^ operating software.

### 3D sphered formation assay

2.0 × 10^3^ cells in 200 μl medium were placed in ULA plates and incubated at 37 °C for 7 days. Next, 100 μl of the medium was gently removed and added 100 μl pre-cold medium containing 3.8 μg/ml of Matrigel (Corning) carefully to avoid bubbles. Cells were kept at 37 °C for 7–10 days. The area of the tumor-spheres was quantified with Image J software.

### Migration and invasion assay

24-well Transwell chambers with 8 mm pore size (Corning Costar) were used to perform the migration and invasion assay. 3 × 10^4^ cells in serum-free medium were inoculated in the upper chamber, whereas the lower chamber was filled with the full condition of medium. Cultures were maintained for 48 h. Afterwards, non-motile cells at the top of the chamber were removed with q-tips and the cells in the bottom chamber were fixed with methanol and stained with DAPI. Five different fields per condition were evaluated using immunofluorescence microscopy. The average number of cells in the five fields per membrane was calculated. Each condition was performed in triplicate. The relative migration/invasion was calculated by the ratio of treated cells to control cells.

### Wound scratch assay

The wound scratch assay was performed as previously described [63].

### Single-molecule fluorescence in situ hybridization (smFISH)

smFISH was conducted as previously described [13]. Images were acquired with a gSTED microscope and spots were counted using the online JAVA software of StarSearch [55]. smFISH probes are listed in Supplementary Table 7.

### RNA antisense purification and mass spectrometry (RAP-MS)

RAP-MS was performed as previously described [13, 26]. Two independent biological replicates were performed. A sample incubated with *Ubiquitin C* (UBC) probes or treated with RNase A (10 ug/ml at 37 °C for 30 min) incubation prior to the hybridization step was used as a negative control to minimize the potential background. 5′-biotinylated 20-mer antisense oligonucleotides are listed in Supplementary Table 7.

### Sequential immunofluorescence and smFISH assay

Cells were grown on coverslips for 48 h and fixed with 4% PFA at room temperature for 10 min, permeabilized with 0.2% Triton X-100/PBS for 5 min at room temperature. Coverslips were then incubated with anti-DHX9 primary antibodies and Alexa Fluor 488-conjugated secondary antibodies and re-fixed with 4% PFA. Next, smFISH was performed as previously described on the same coverslips. Digital photographs were acquired with a Two-Photon Excitation gSTED Microscope (Leica) and visualized in Leica Advanced Fluorescence software (Leica).

### Animal studies

The animal experiments were approved by the Animal Ethics Committee of Cancer Research UK Manchester Institute under the Animal Scientific Procedures Act 1986 and according to the ARRIVE guidelines and the Committee of the National Cancer Research Institute guidelines. All mice were initially blinding randomized to different groups and were maintained in a pathogen-free environment with free access to food/water. Treatments, measurements and dissection were performed by Biological Resource Unit (BRU) colleagues thoroughly. Mice were observed for signs of illness or distress during the experiments and body weight and tumor sizes were measured twice a week. Animals were euthanized after the appearance of predefined criteria like rapid weight loss (>20%) or weight gain (>20% due to ascites) and labored respiration. After euthanasia, lungs, liver and kidneys were excised, weighed, photographed and bisected. Organs were fixed in formalin immediately and subjected to Haematoxylin Staining (HE) and immunohistochemistry (IHC) for further analysis.

### Murine subcutaneous in vivo model

H1299 and H460 cells (5 × 10^6^) stably expressing a control vector (Ev) or *HIF1A-As2* were subcutaneously injected into the right posterior dorsal flank of 4–6 weeks old female NOD/SCID Gamma (NSG) mice (Charles River). All animals were maintained in a pathogen-free environment with free access to food and water. Tumor size was assessed twice per week with a digital calliper to measure the length (*l*) and the width (*w*) and calculated based on the formula *V* = *lw*^2^/2. Mice were euthanized and sacrificed when tumor size reached the endpoint of 1500 mm^3^ or mice displayed signs of illness.

### Metastasis mouse model

Female NSG mice (4–6 weeks old) in each experimental group were injected with 5 × 10^6^ H1299-*HIF1A-As2* or H1299-Ev cells in 0.2 ml PBS through the tail vein (*n* = 7). Seventeen weeks later, mice were sacrificed, and organs were collected for further analysis.

### Orthotopic mouse model

NSG mice were anaesthetized with isoflurane and placed in the right lateral decubitus position. H1299 or H460 cells (2.5 × 10^6^) stably expressing *HIF1A-As2* or an empty vector, or H1299 DHX9 KO or control (Cas9) cells using the CRISPR/Cas9 editing system were injected with a 0.5 ml syringe percutaneously into the left lateral thorax, at the lateral dorsal axillary line of 4–6 weeks old NSG mice. After the injection, mice were transferred to a clean recovery cage on top of a heated mat and observed until they fully recovered. Mice were sacrificed 5 weeks after the injection of the cells. Lungs, livers and kidneys were collected after autopsy for histological analysis.

### KRAS^LSLG12D^ mouse model

KRAS^LSLG12D^ mice were divided in 3 groups. Group 2 and 3 received adenoCRE (1 × 10^7^ PFU in 50 μl PBS) recombinase by intranasal inhalation at 6 weeks of age. 5 weeks after adenoCRE administration mice in group 2 were treated with ASO Ctrl (20 mg/kg) and mice in group 3 with ASO#1 (20 mg/kg) once per week for seven weeks. 5 mg/kg mice of cisplatin will be given to the mice intraperitoneally (i.p.) twice during the entire course of the experiment (at week 7 and week 10 after CRE inhalation). 12 weeks after initial adenoCRE inhalation mice were euthanized, lungs were weighted, tumors and normal lungs were harvested, and blood was collected for analysis.

### Patient-derived xenograft (PDX) mouse model

PDX was performed as previously described [13]. IC11LC13 sample, from a patient with metastatic lung cancer, was transplanted into the right posterior dorsal flank of 4–6 weeks old female NSG mice (*n* = 4 per group). Mice were intravenously injected with vehicle (Ctrl), 10058-F4 (15 mg/kg), *HIF1A-As2* ASO#1 (20 mg/kg) three times per week (the first 2 weeks) and then twice a week (the last 2 weeks) for a total of 10 injections. All mice were weighed twice a week. Tumor growth was monitored by measuring tumor diameters with a calliper. Mice were euthanized and sacrificed when tumor size reached the endpoint of 1500 mm^3^ or mice displayed signs of illness.

### Statistical analysis

All data are presented as the mean value of ±S.D (*n* = 3) otherwise indicated differently. The significances were calculated by two paired *t-*tests. *p < 0.05 and **p < 0.001 were defined as statistically significant. Pearson’s correlation was calculated using the GraphPad Prism package (GraphPad Software Inc.).

## Supplementary information

Supplementary Figure 1

Supplementary Figure 2

Supplementary Figure 3

Supplementary Figure 4

Supplementary Figure 5

Supplementary Figure 6

Supplementary Figure 7

Supplementary Figure 8

Supplementary Figure 9

Supplementary Figure 10

Supplementary Figure 11

Supplementary Figure 12

Supplementary Figure 13

Supplementary Figure 14

Supplementary Figure 15

Checklist

Supplementary Figure Legend

Supplementary Table 1. Proteins interacting with HIF1A-As2 by RAP-MS

Supplementary Table 2. PCR Primers

Supplementary Table 3. qPCR primers and probes

Supplementary Table 4. Antibodies

Supplementary Table 5. siRNAs and ASOs

Supplementary Table 6. Plasmid

Supplementary Table 7. lncRNA smFISH and RAP-MS probes

## Data Availability

RNA-seq and ChIP-seq data that supports the findings of this study have been deposited in the Gene Expression Omnibus under the accession code: GSE124626, GSE124627, GSE124628 and GSE124630, respectively. Publicly available ChIP-seq data are available from ENCODE (https://www.encodeproject.org). The mass spectrometry proteomics data have been deposited to the ProteomeXchange Consortium via the PRIDE partner repository with the dataset identifier PXD024388 and link 10.6019/PXD024388. A list of proteins interacting with *HIF1A-As2* by mass spectrometry is provided in Supplementary Table 1. All other data supporting the findings of this study are available from the corresponding author.

## References

[1] Ferlay J, Soerjomataram I, Dikshit R, Eser S, Mathers C, Rebelo M (2015). Cancer incidence and mortality worldwide: sources, methods and major patterns in GLOBOCAN 2012. Int J Cancer.

[2] Sung H, Ferlay J, Siegel RL, Laversanne M, Soerjomataram I, Jemal A (2021). Global Cancer Statistics 2020: GLOBOCAN Estimates of Incidence and Mortality Worldwide for 36 Cancers in 185 Countries. CA Cancer J Clin.

[3] Chen Z, Fillmore CM, Hammerman PS, Kim CF, Wong KK (2014). Non-small-cell lung cancers: a heterogeneous set of diseases. Nat Rev Cancer.

[4] Shi L, Middleton J, Jeon YJ, Magee P, Veneziano D, Lagana A (2018). KRAS induces lung tumorigenesis through microRNAs modulation. Cell Death Dis.

[5] Vigil D, Cherfils J, Rossman KL, Der CJ (2010). Ras superfamily GEFs and GAPs: validated and tractable targets for cancer therapy?. Nat Rev Cancer.

[6] Xue JY, Zhao Y, Aronowitz J, Mai TT, Vides A, Qeriqi B (2020). Rapid non-uniform adaptation to conformation-specific KRAS(G12C) inhibition. Nature.

[7] Canon J, Rex K, Saiki AY, Mohr C, Cooke K, Bagal D (2019). The clinical KRAS(G12C) inhibitor AMG 510 drives anti-tumour immunity. Nature.

[8] Quinn JJ, Chang HY (2016). Unique features of long non-coding RNA biogenesis and function. Nat Rev Genet.

[9] Ransohoff JD, Wei Y, Khavari PA (2018). The functions and unique features of long intergenic non-coding RNA. Nat Rev Mol Cell Biol.

[10] Schmitt AM, Chang HY (2016). Long Noncoding RNAs in Cancer Pathways. Cancer Cell.

[11] Loewen G, Jayawickramarajah J, Zhuo Y, Shan B (2014). Functions of lncRNA HOTAIR in lung cancer. J Hematol Oncol.

[12] Yu T, Zhao Y, Hu Z, Li J, Chu D, Zhang J (2017). MetaLnc9 Facilitates Lung Cancer Metastasis via a PGK1-Activated AKT/mTOR Pathway. Cancer Res.

[13] Shi L, Magee P, Fassan M, Sahoo S, Leong HS, Lee D (2021). A KRAS-responsive long non-coding RNA controls microRNA processing. Nat Commun.

[14] Corcoran SE, O’Neill LA (2016). HIF1alpha and metabolic reprogramming in inflammation. J Clin Investig.

[15] Lin J, Shi Z, Yu Z, He Z (2018). LncRNA *HIF1A-AS2* positively affects the progression and EMT formation of colorectal cancer through regulating miR-129-5p and DNMT3A. Biomed Pharmacother.

[16] Wang Y, Zhang G, Han J (2019). *HIF1A-AS2* predicts poor prognosis and regulates cell migration and invasion in triple-negative breast cancer. J Cell Biochem.

[17] Mineo M, Ricklefs F, Rooj AK, Lyons SM, Ivanov P, Ansari KI (2016). The Long Non-coding RNA *HIF1A-AS2* Facilitates the Maintenance of Mesenchymal Glioblastoma Stem-like Cells in Hypoxic Niches. Cell Rep.

[18] Molina-Arcas M, Hancock DC, Sheridan C, Kumar MS, Downward J (2013). Coordinate direct input of both KRAS and IGF1 receptor to activation of PI3 kinase in KRAS-mutant lung cancer. Cancer Discov.

[19] Uprety D, Adjei AA (2020). KRAS: From undruggable to a druggable Cancer Target. Cancer Treat Rev.

[20] Amodio N, Stamato MA, Juli G, Morelli E, Fulciniti M, Manzoni M (2018). Drugging the lncRNA MALAT1 via LNA gapmeR ASO inhibits gene expression of proteasome subunits and triggers anti-multiple myeloma activity. Leukemia.

[21] Tay Y, Rinn J, Pandolfi PP (2014). The multilayered complexity of ceRNA crosstalk and competition. Nature.

[22] Li J, Ma W, Zeng P, Wang J, Geng B, Yang J (2015). LncTar: a tool for predicting the RNA targets of long noncoding RNAs. Brief Bioinform.

[23] Hur K, Toiyama Y, Takahashi M, Balaguer F, Nagasaka T, Koike J (2013). MicroRNA-200c modulates epithelial-to-mesenchymal transition (EMT) in human colorectal cancer metastasis. Gut.

[24] Long Y, Wang X, Youmans DT, Cech TR (2017). How do lncRNAs regulate transcription. Sci Adv.

[25] Engreitz JM, Pandya-Jones A, McDonel P, Shishkin A, Sirokman K, Surka C (2013). The Xist lncRNA exploits three-dimensional genome architecture to spread across the X chromosome. Science.

[26] McHugh CA, Chen CK, Chow A, Surka CF, Tran C, McDonel P (2015). The Xist lncRNA interacts directly with SHARP to silence transcription through HDAC3. Nature.

[27] Lee T, Pelletier J (2016). The biology of DHX9 and its potential as a therapeutic target. Oncotarget.

[28] Fuller-Pace FV (2006). DExD/H box RNA helicases: multifunctional proteins with important roles in transcriptional regulation. Nucleic Acids Res.

[29] Ding X, Jia X, Wang C, Xu J, Gao SJ, Lu C (2019). A DHX9-lncRNA-MDM2 interaction regulates cell invasion and angiogenesis of cervical cancer. Cell Death Differ.

[30] Kopp F, Mendell JT (2018). Functional Classification and Experimental Dissection of Long Noncoding RNAs. Cell.

[31] Stine ZE, Walton ZE, Altman BJ, Hsieh AL, Dang CVMYC (2015). Metabolism, and Cancer. Cancer Discov.

[32] Rouillard AD, Gundersen GW, Fernandez NF, Wang Z, Monteiro CD, McDermott MG (2016). The harmonizome: a collection of processed datasets gathered to serve and mine knowledge about genes and proteins. Database (Oxford).

[33] Chu C, Quinn J, Chang HY. Chromatin isolation by RNA purification (ChIRP). J Vis Exp. 2012;3912. 10.3791/3912 PMC3460573 22472705

[34] Dang CV (2012). MYC on the path to cancer. Cell.

[35] Meyer N, Penn LZ (2008). Reflecting on 25 years with MYC. Nat Rev Cancer.

[36] Schmidt S, Gay D, Uthe FW, Denk S, Paauwe M, Matthes N (2019). A MYC-GCN2-eIF2alpha negative feedback loop limits protein synthesis to prevent MYC-dependent apoptosis in colorectal cancer. Nat Cell Biol.

[37] Wang Z, Yang B, Zhang M, Guo W, Wu Z, Wang Y (2018). lncRNA Epigenetic Landscape Analysis Identifies EPIC1 as an Oncogenic lncRNA that Interacts with MYC and Promotes Cell-Cycle Progression in Cancer. Cancer Cell.

[38] Jung P, Menssen A, Mayr D, Hermeking H (2008). AP4 encodes a c-MYC-inducible repressor of p21. Proc Natl Acad Sci USA.

[39] Sun H, Lin DC, Cao Q, Pang B, Gae DD, Lee VKM (2017). Identification of a Novel SYK/c-MYC/MALAT1 Signaling Pathway and Its Potential Therapeutic Value in Ewing Sarcoma. Clin Cancer Res.

[40] Bailey TL, Boden M, Buske FA, Frith M, Grant CE, Clementi L (2009). MEME SUITE: tools for motif discovery and searching. Nucleic Acids Res.

[41] Mu L, Wang Y, Su H, Lin Y, Sui W, Yu X (2021). *HIF1A-AS2* Promotes the Proliferation and Metastasis of Gastric Cancer Cells Through miR-429/PD-L1 Axis. Dig Dis Sci.

[42] Chen M, Wei X, Shi X, Lu L, Zhang G, Huang Y (2021). LncRNA *HIF1A-AS2* accelerates malignant phenotypes of renal carcinoma by modulating miR-30a-5p/SOX4 axis as a ceRNA. Cancer Biol Med.

[43] Wang X, Peng L, Gong X, Zhang X, Sun R (2019). LncRNA *HIF1A-AS2* promotes osteosarcoma progression by acting as a sponge of miR-129-5p. Aging (Albany NY).

[44] Si J, Ma Y, Lv C, Hong Y, Tan H, Yang Y (2021). *HIF1A-AS2* induces osimertinib resistance in lung adenocarcinoma patients by regulating the miR-146b-5p/IL-6/STAT3 axis. Mol Ther Nucleic Acids.

[45] Guclu E, Eroglu Gunes C, Kurar E, Vural H (2021). Knockdown of lncRNA *HIF1A-AS2* increases drug sensitivity of SCLC cells in association with autophagy. Med Oncol.

[46] Wang YL, Liu JY, Yang JE, Yu XM, Chen ZL, Chen YJ (2019). Lnc-UCID Promotes G1/S Transition and Hepatoma Growth by Preventing DHX9-Mediated CDK6 Down-regulation. Hepatology.

[47] Bretones G, Delgado MD, Leon J (2015). Myc and cell cycle control. Biochim Biophys Acta.

[48] Baluapuri A, Wolf E, Eilers M (2020). Target gene-independent functions of MYC oncoproteins. Nat Rev Mol Cell Biol.

[49] Damase TR, Sukhovershin R, Boada C, Taraballi F, Pettigrew RI, Cooke JP (2021). The Limitless Future of RNA Therapeutics. Front Bioeng Biotechnol.

[50] Garbo S, Maione R, Tripodi M, Battistelli C (2022). Next RNA Therapeutics: The Mine of Non-Coding. Int J Mol Sci.

[51] Kim YK (2022). RNA therapy: rich history, various applications and unlimited future prospects. Exp Mol Med.

[52] Wang Y, Zhu P, Luo J, Wang J, Liu Z, Wu W (2019). LncRNA HAND2-AS1 promotes liver cancer stem cell self-renewal via BMP signaling. EMBO J.

[53] Arun G, Diermeier S, Akerman M, Chang KC, Wilkinson JE, Hearn S (2016). Differentiation of mammary tumors and reduction in metastasis upon Malat1 lncRNA loss. Genes Dev.

[54] Zhu P, Wu J, Wang Y, Zhu X, Lu T, Liu B (2018). LncGata6 maintains stemness of intestinal stem cells and promotes intestinal tumorigenesis. Nat Cell Biol.

[55] Torre E, Dueck H, Shaffer S, Gospocic J, Gupte R, Bonasio R (2018). Rare Cell Detection by Single-Cell RNA Sequencing as Guided by Single-Molecule RNA FISH. Cell Syst.

[56] Bankhead P, Loughrey MB, Fernandez JA, Dombrowski Y, McArt DG, Dunne PD (2017). QuPath: Open source software for digital pathology image analysis. Sci Rep.

[57] Abugessaisa I, Ramilowski JA, Lizio M, Severin J, Hasegawa A, Harshbarger J (2021). FANTOM enters 20th year: expansion of transcriptomic atlases and functional annotation of non-coding RNAs. Nucleic Acids Res.

[58] Lee TI, Johnstone SE, Young RA (2006). Chromatin immunoprecipitation and microarray-based analysis of protein location. Nat Protoc.

[59] Liao Y, Smyth GK, Shi W (2013). The Subread aligner: fast, accurate and scalable read mapping by seed-and-vote. Nucleic Acids Res.

[60] Love MI, Huber W, Anders S (2014). Moderated estimation of fold change and dispersion for RNA-seq data with DESeq2. Genome Biol.

[61] Subramanian A, Tamayo P, Mootha VK, Mukherjee S, Ebert BL, Gillette MA (2005). Gene set enrichment analysis: a knowledge-based approach for interpreting genome-wide expression profiles. Proc Natl Acad Sci U S A.

[62] Ramanathan M, Porter DF, Khavari PA (2019). Methods to study RNA-protein interactions. Nat Methods.

[63] Shi L, Jackstadt R, Siemens H, Li H, Kirchner T, Hermeking H (2014). p53-induced miR-15a/16-1 and AP4 form a double-negative feedback loop to regulate epithelial-mesenchymal transition and metastasis in colorectal cancer. Cancer Res.

