# Supplementary material for: Long non-coding RNA HIF1A-As2 and MYC form a double-positive feedback loop to promote cell proliferation and metastasis in KRAS-driven non-small cell lung cancer
Source: Cell Death Differ. 2023 Apr 11;30(6):1533–49. doi: 10.1038/s41418-023-01160-x (PMC10089381; doi:10.1038/s41418-023-01160-x)

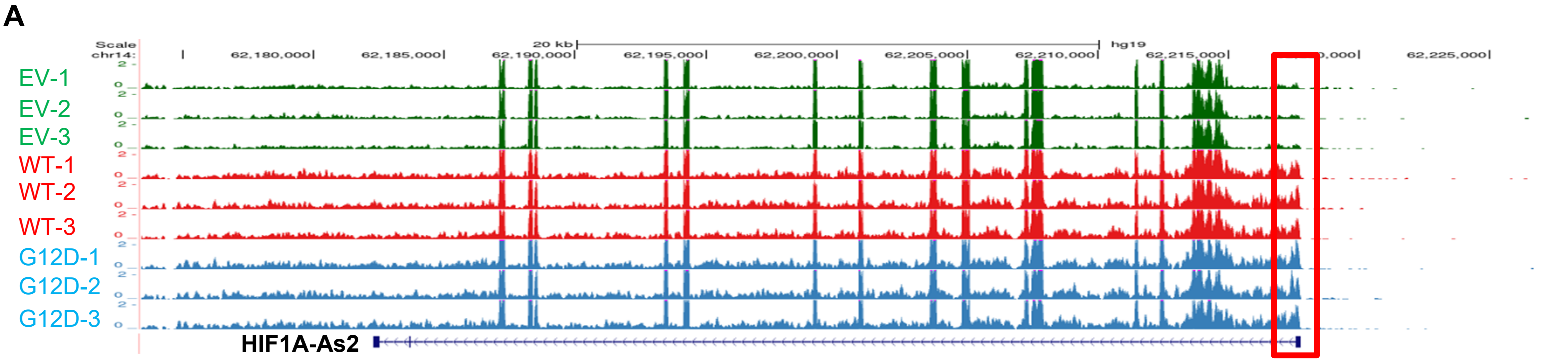

B

Algorithms	Raw results	Interpretation
PRIDE reprocessing 2.0	0	Non-coding
Lee translation initiation sites	0	Non-coding
PhyloCSF score	-42.3452	Non-coding
CPAT coding probability	1.41%	Non-coding
Bazzini small ORFs	0	Non-coding

C

Locus conservation	HIF1A-As2
Chimp	No
Mouse	No
Fish	No
Fly	Yes

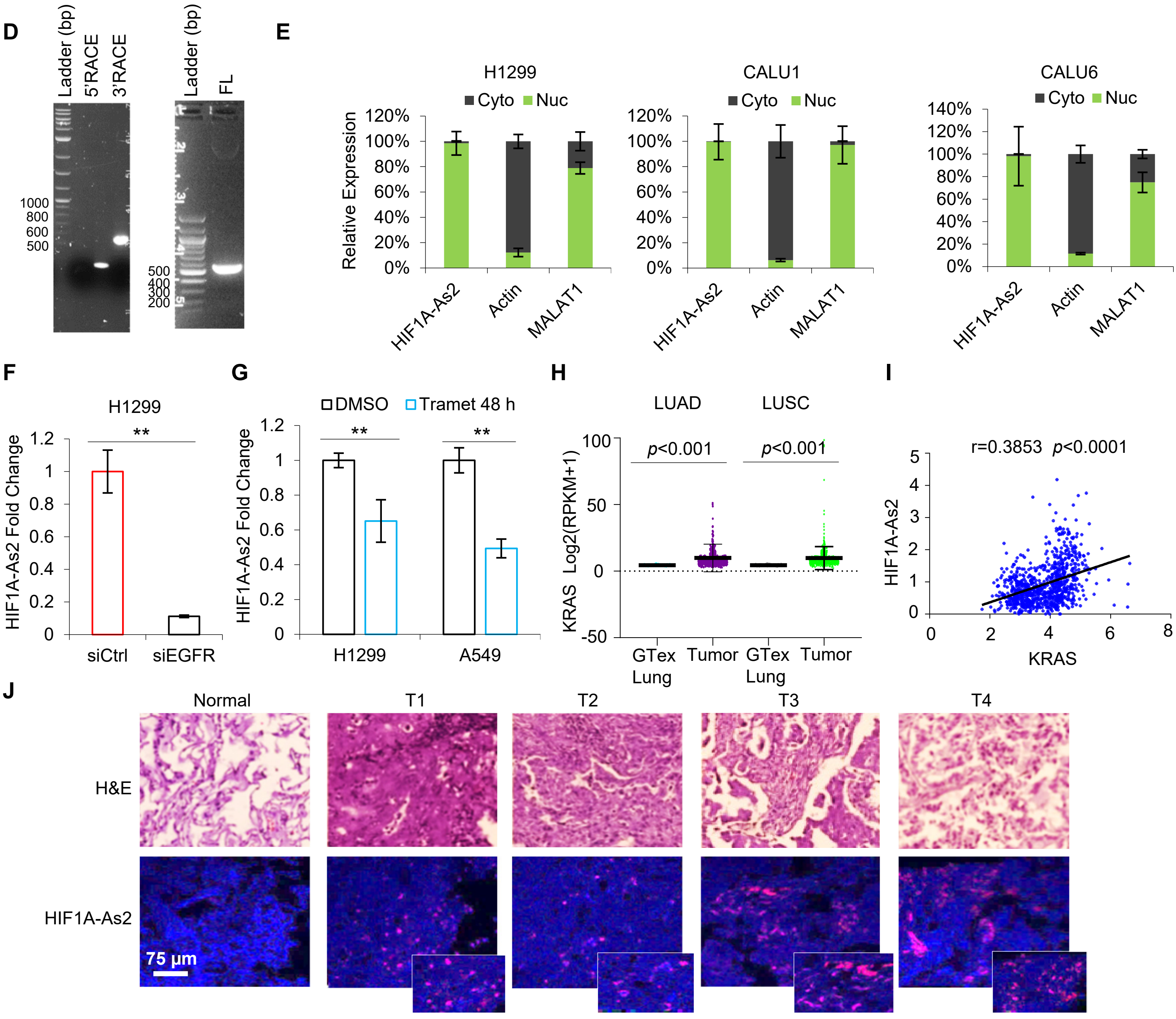

Supplement: Supplementary file 1 — Supplementary Figure 1 [file 41418_2023_1160_MOESM1_ESM.pdf]
