# Supplementary material for: Long non-coding RNA HIF1A-As2 and MYC form a double-positive feedback loop to promote cell proliferation and metastasis in KRAS-driven non-small cell lung cancer
Source: Cell Death Differ. 2023 Apr 11;30(6):1533–49. doi: 10.1038/s41418-023-01160-x (PMC10089381; doi:10.1038/s41418-023-01160-x)

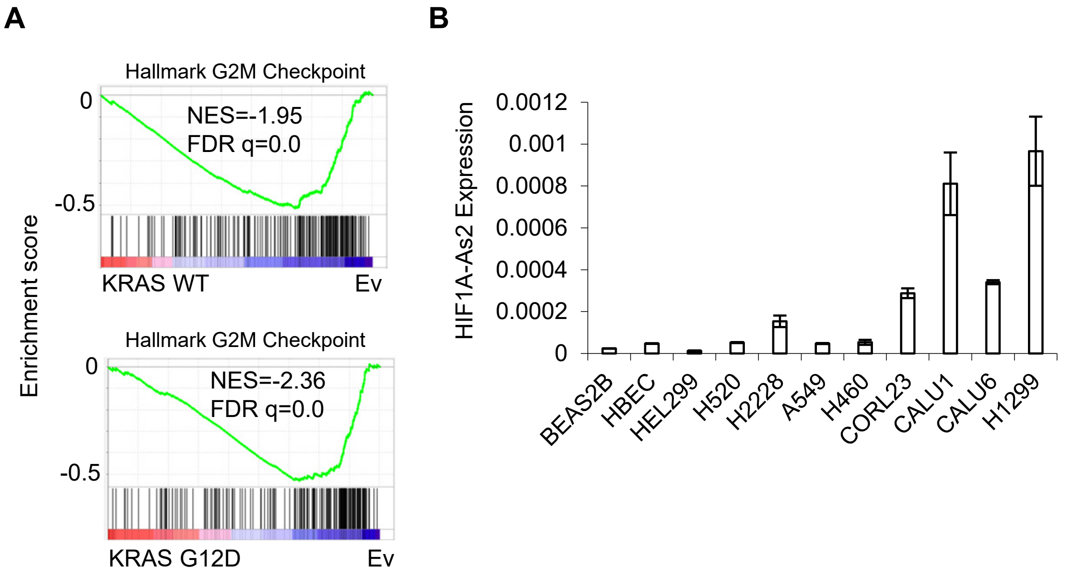

Cell line	KRAS status
BEAS2B	KRAS WT
HBEC	KRAS WT
HEL299	KRAS WT
H520	KRAS WT
H2228	KRAS WT
A549	KRAS G12S
H460	KRAS Q61H
CORL-23	KRAS WT
CALU1	KRAS G12C
CALU6	KRAS Q61K
H1299	KRAS WT

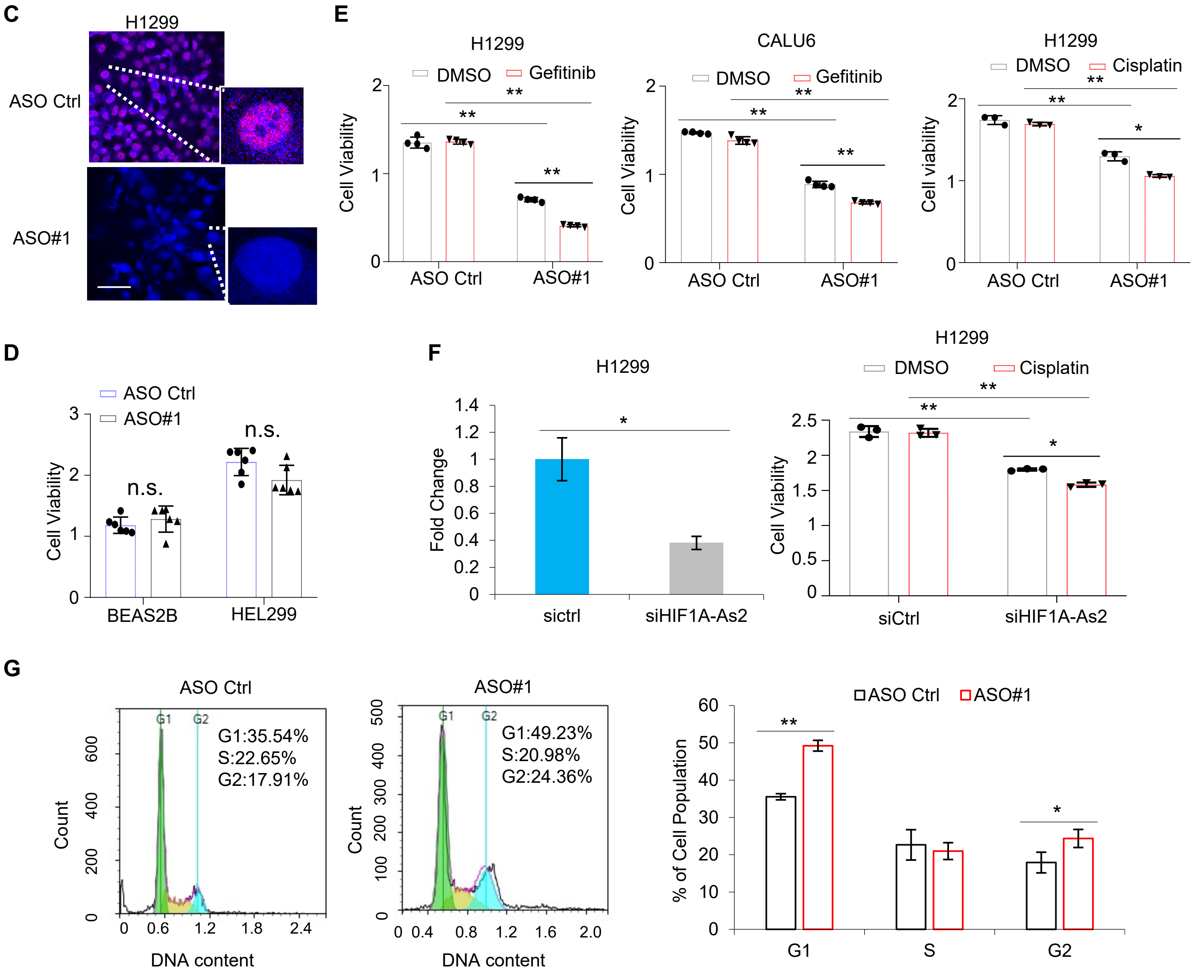

Supplement: Supplementary file 2 — Supplementary Figure 2 [file 41418_2023_1160_MOESM2_ESM.pdf]
