# Supplementary figures and images for: Long non-coding RNA HIF1A-As2 and MYC form a double-positive feedback loop to promote cell proliferation and metastasis in KRAS-driven non-small cell lung cancer
Source: Cell Death Differ. 2023 Apr 11;30(6):1533–49. doi: 10.1038/s41418-023-01160-x (PMC10089381; doi:10.1038/s41418-023-01160-x)

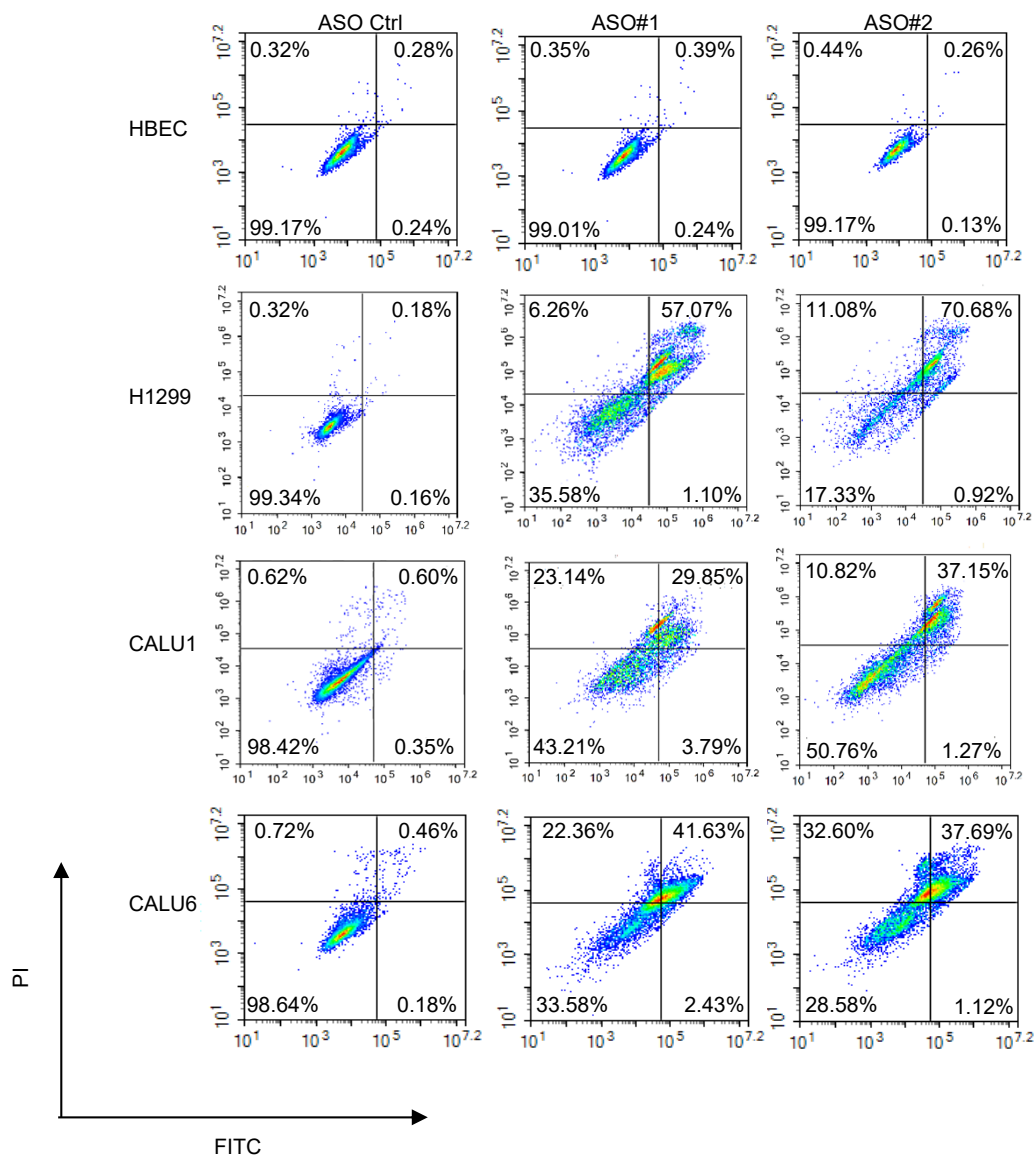

Supplement: Supplementary file 3 — Supplementary Figure 3 [file 41418_2023_1160_MOESM3_ESM.pdf]

**A**

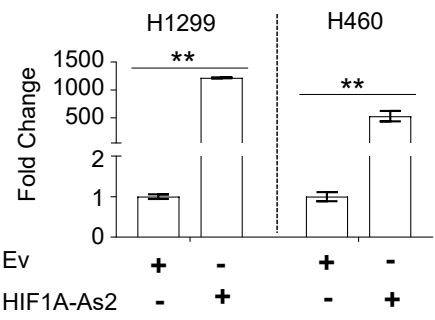

**B**

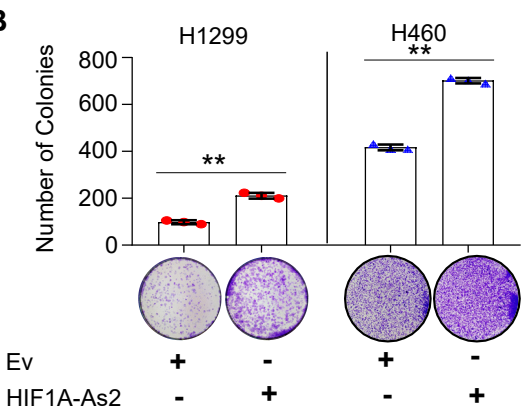

**C**

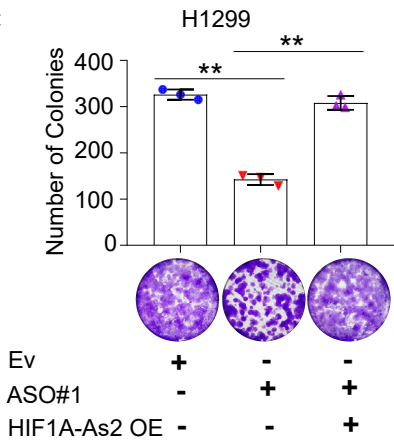

Supplement: Supplementary file 4 — Supplementary Figure 4 [file 41418_2023_1160_MOESM4_ESM.pdf]

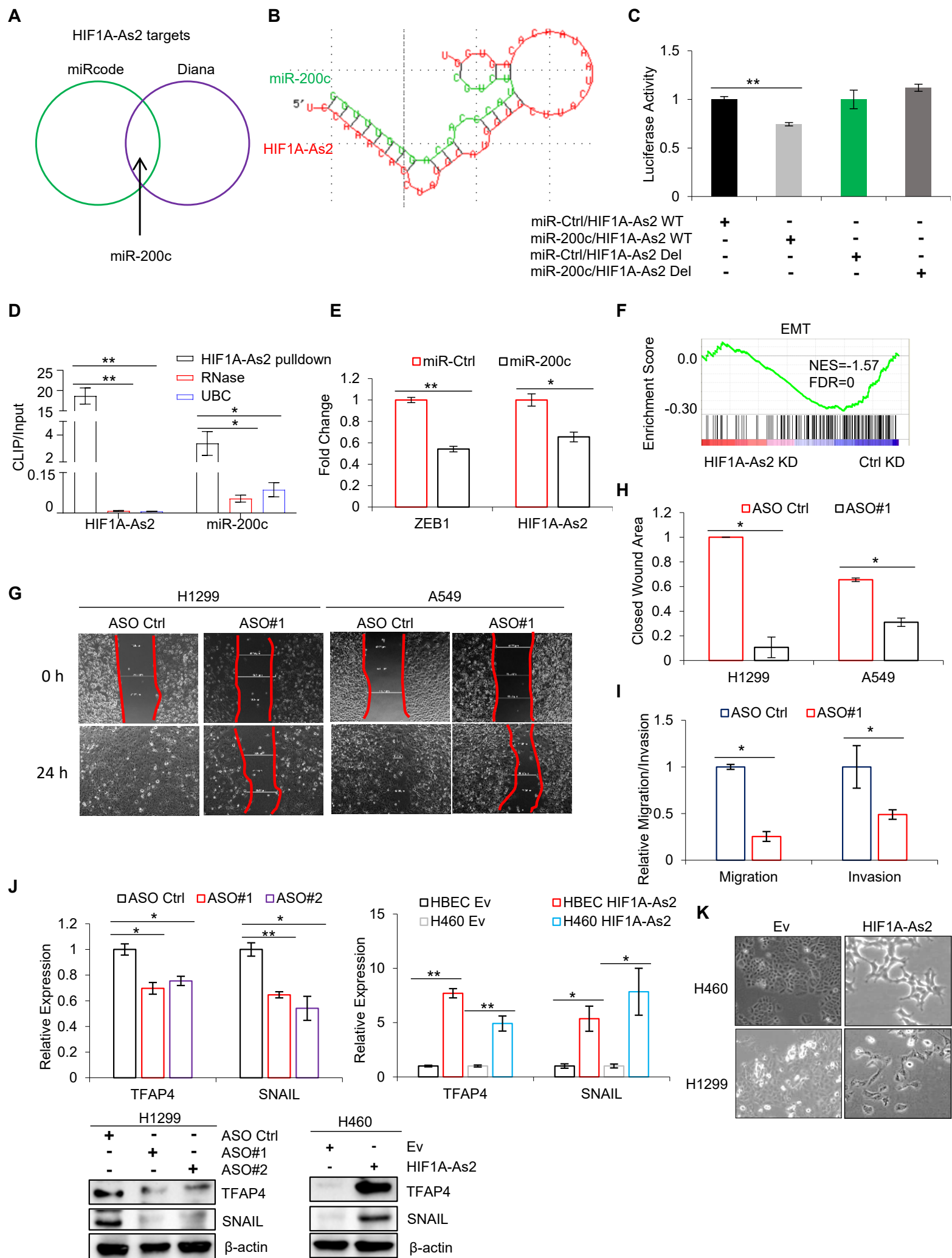

Supplement: Supplementary file 5 — Supplementary Figure 5 [file 41418_2023_1160_MOESM5_ESM.pdf]

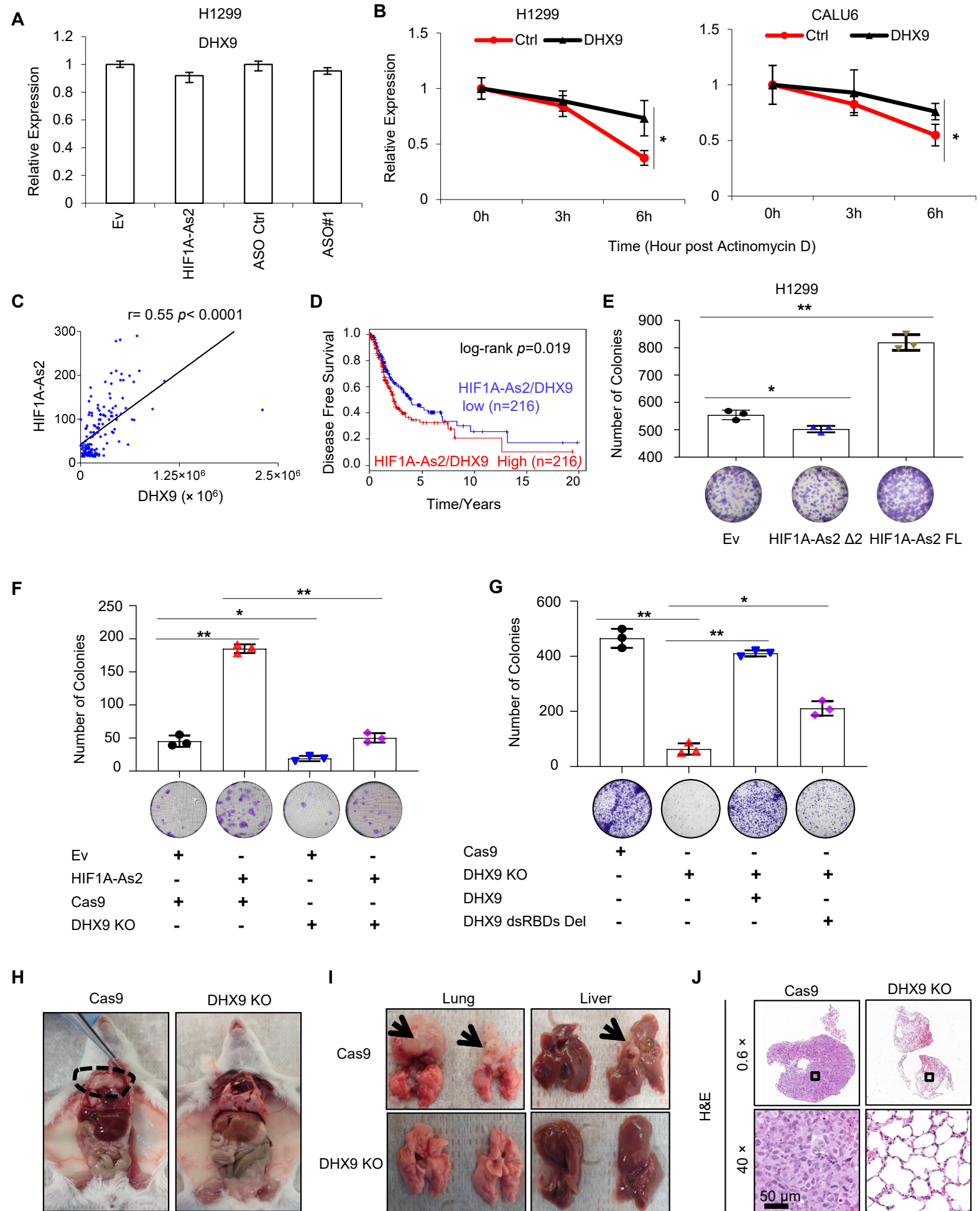

Supplement: Supplementary file 7 — Supplementary Figure 7 [file 41418_2023_1160_MOESM7_ESM.pdf]

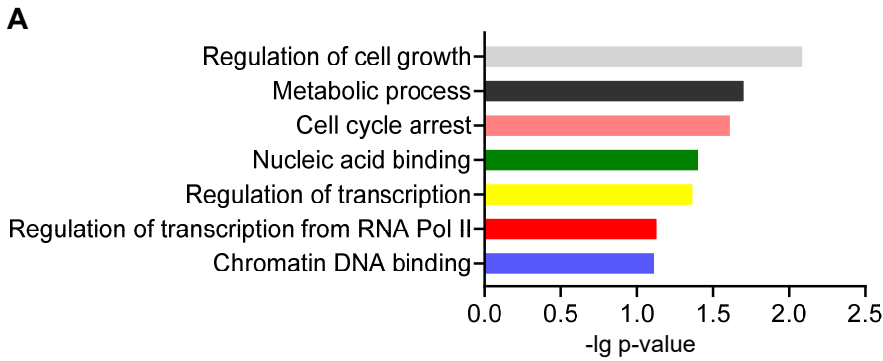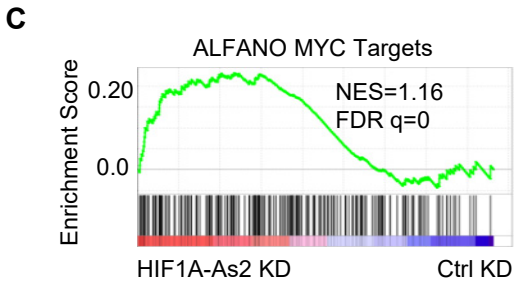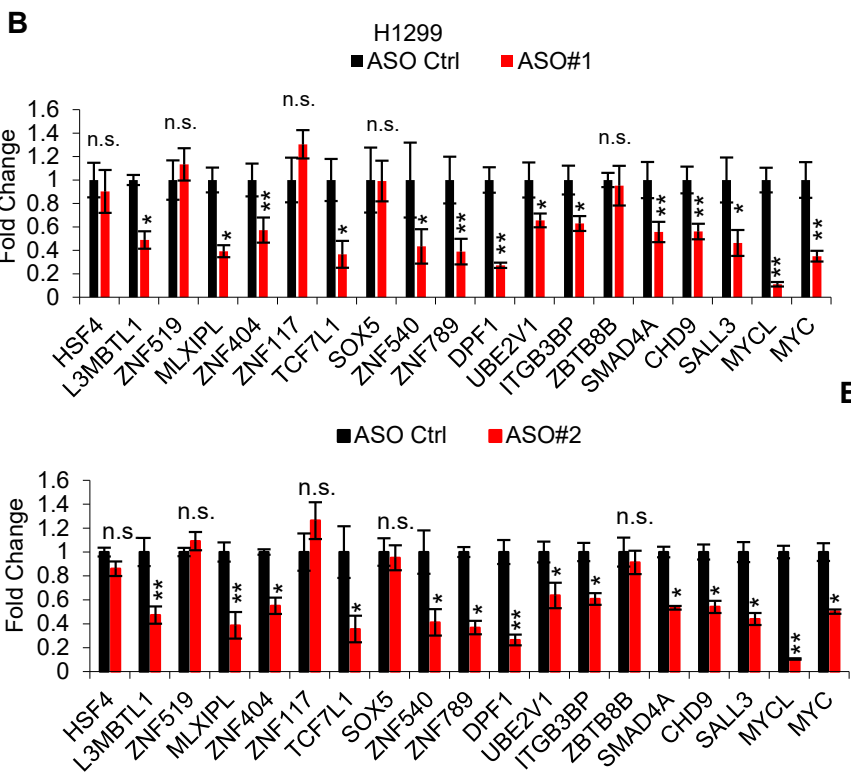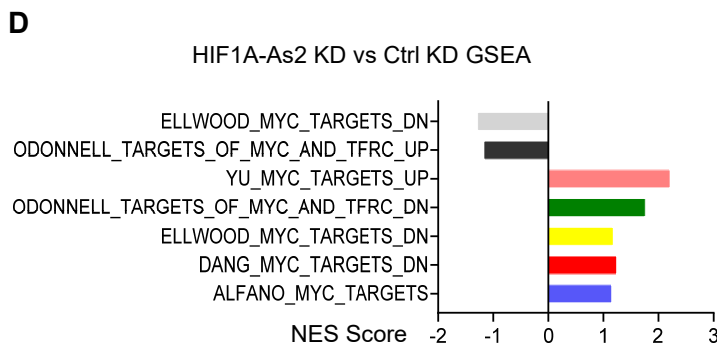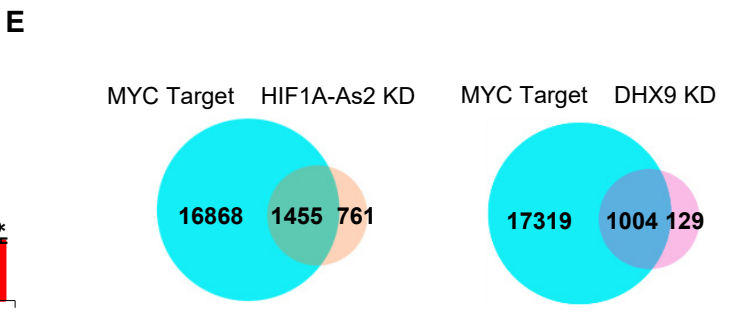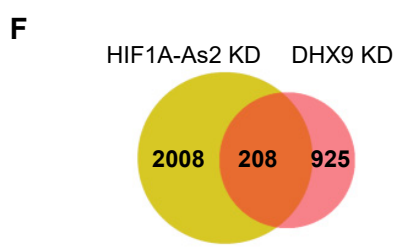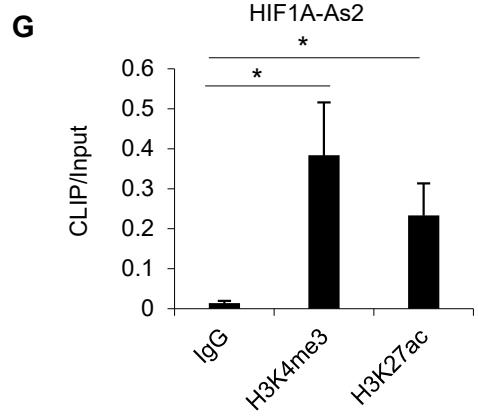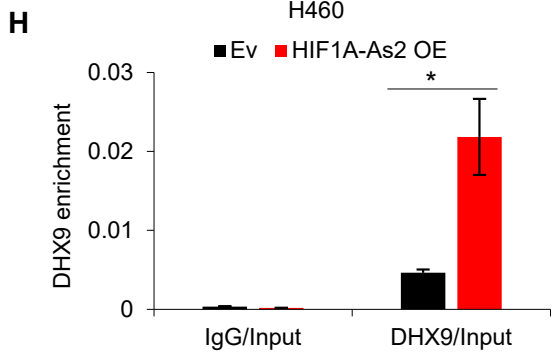

Supplement: Supplementary file 8 — Supplementary Figure 8 [file 41418_2023_1160_MOESM8_ESM.pdf]

**A**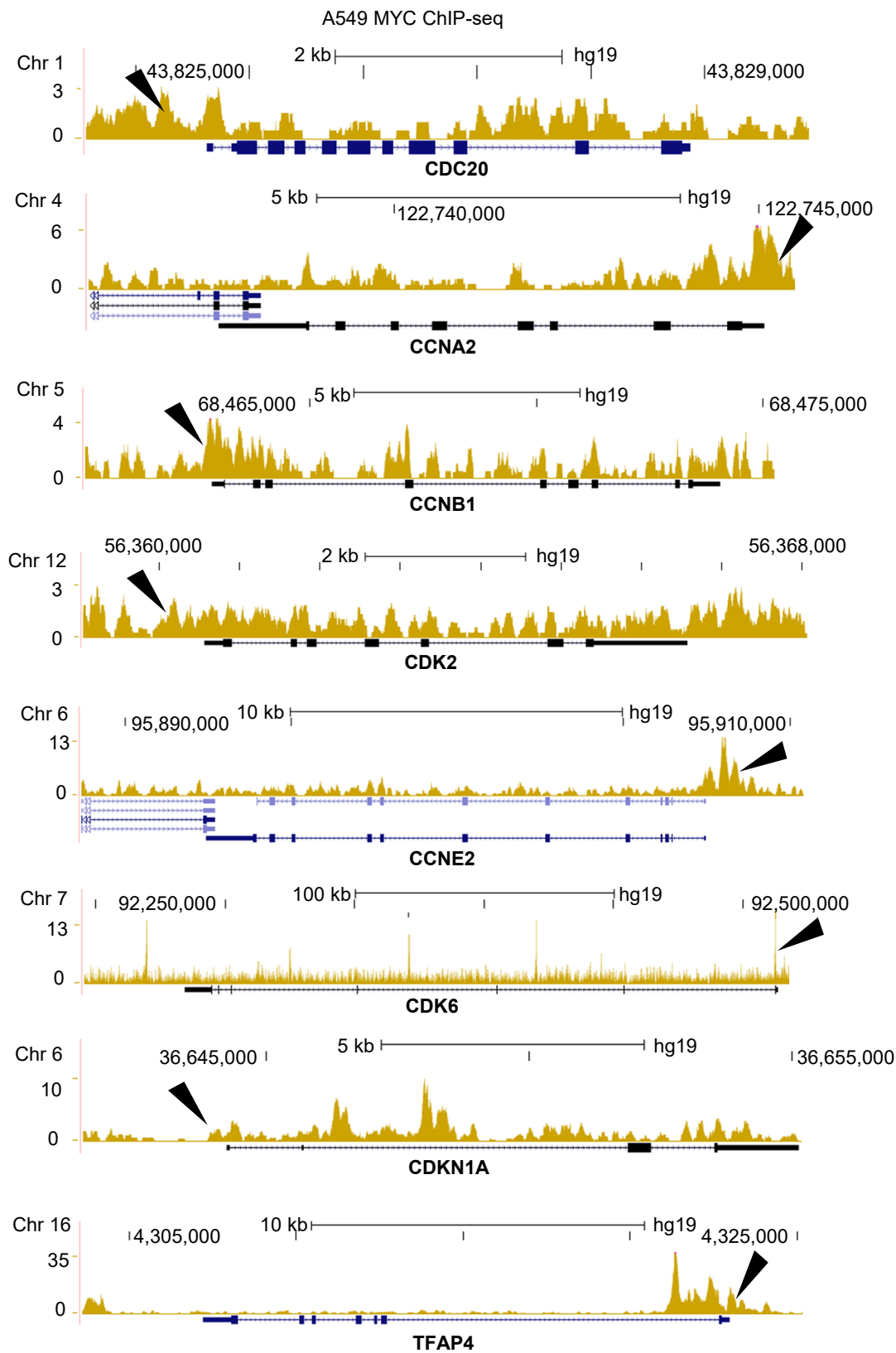**B**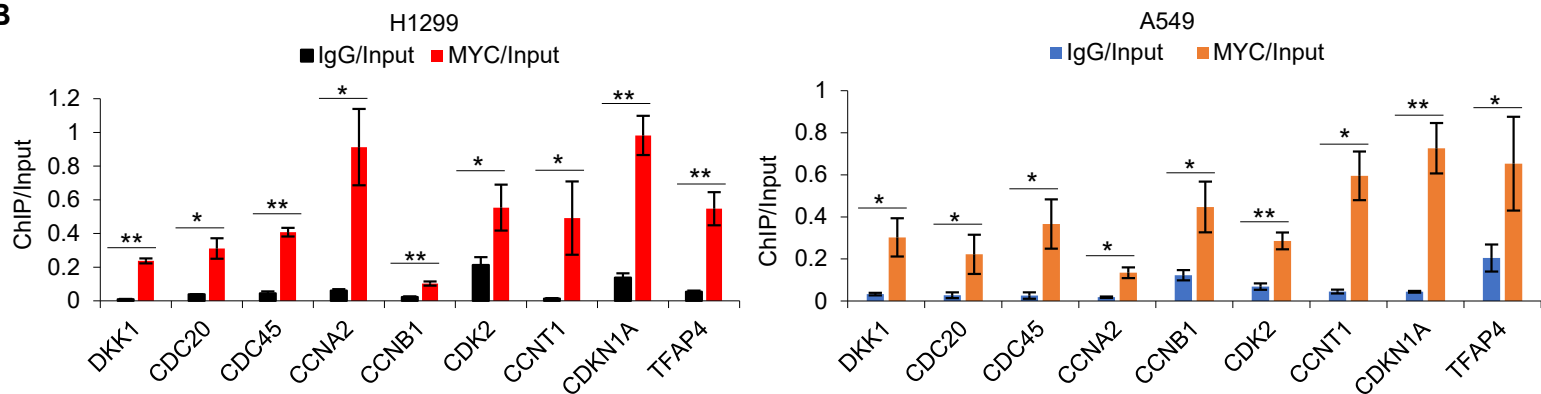

Supplement: Supplementary file 9 — Supplementary Figure 9 [file 41418_2023_1160_MOESM9_ESM.pdf]

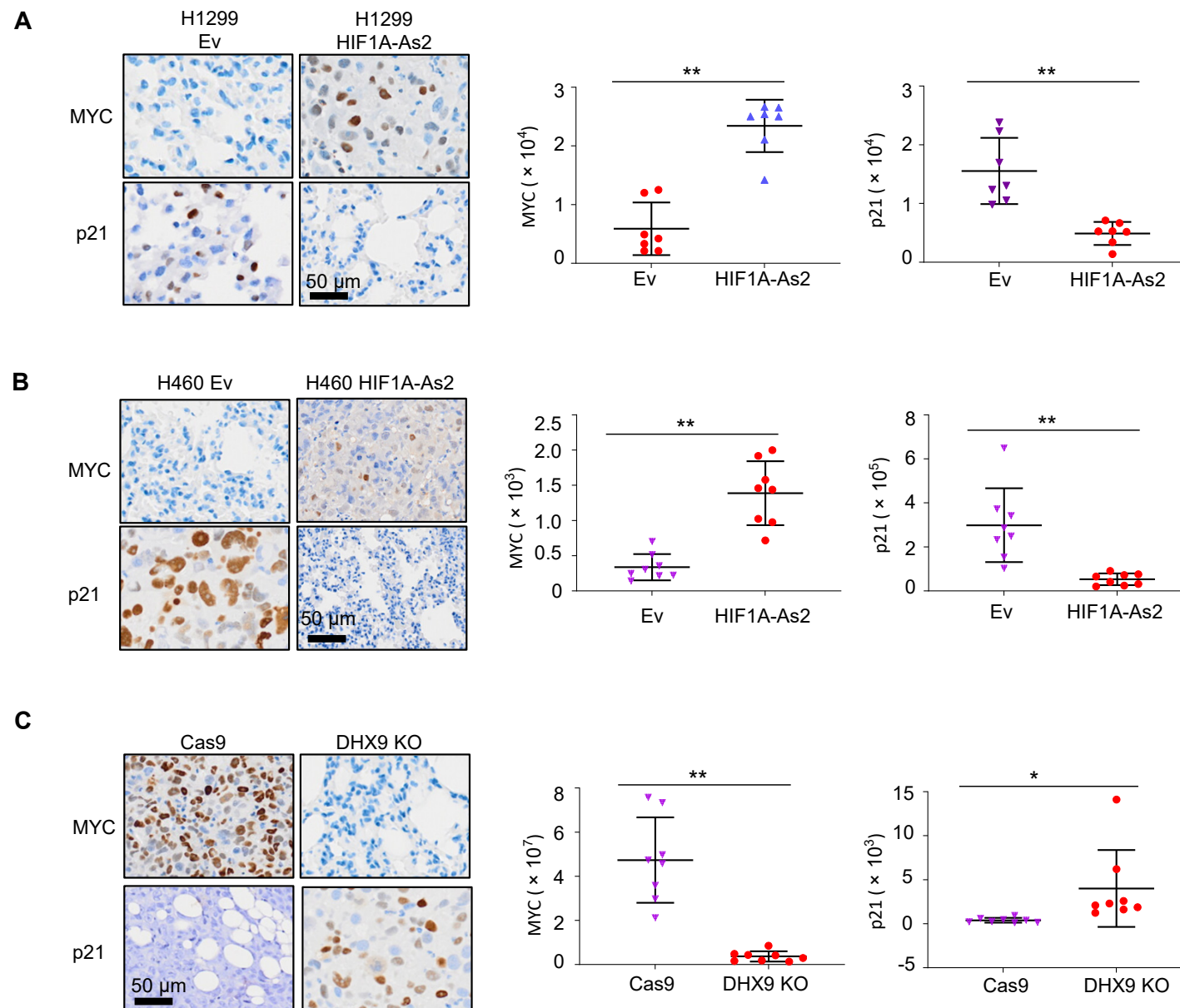

Supplement: Supplementary file 10 — Supplementary Figure 10 [file 41418_2023_1160_MOESM10_ESM.pdf]

**A**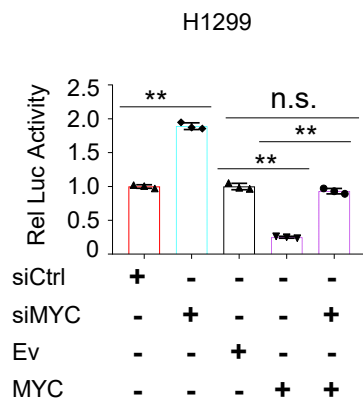**B**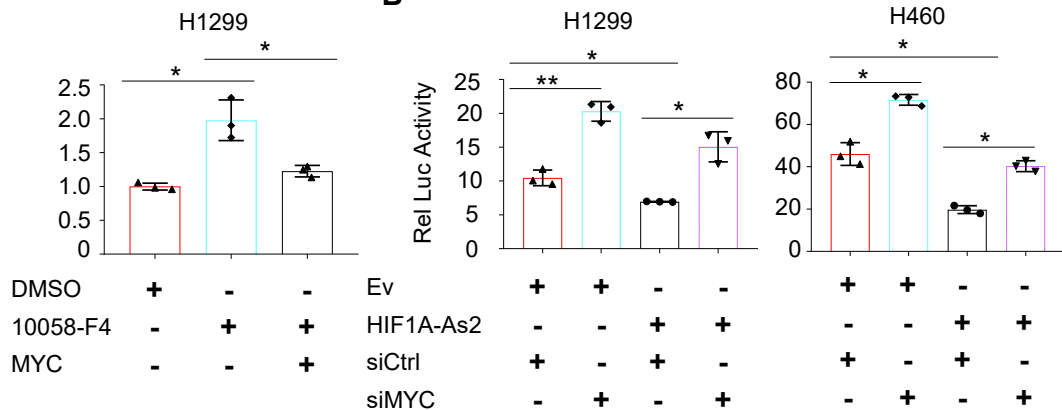**C**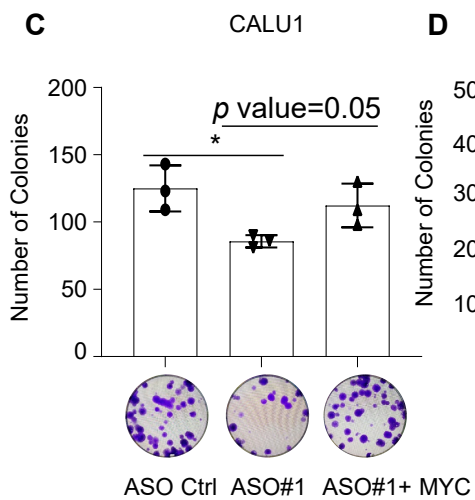**D**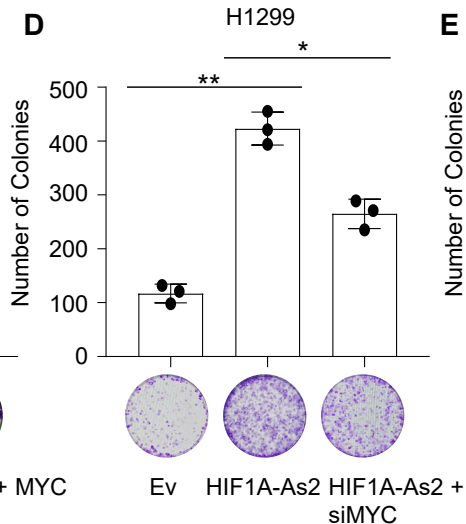**E**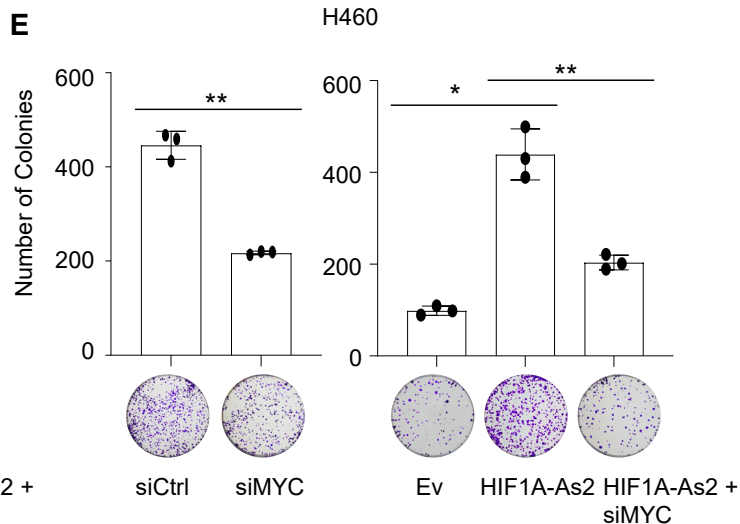

Supplement: Supplementary file 11 — Supplementary Figure 11 [file 41418_2023_1160_MOESM11_ESM.pdf]

**A**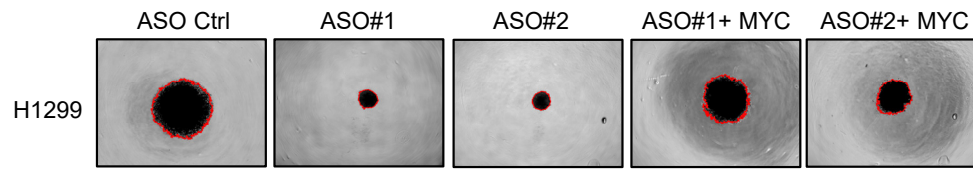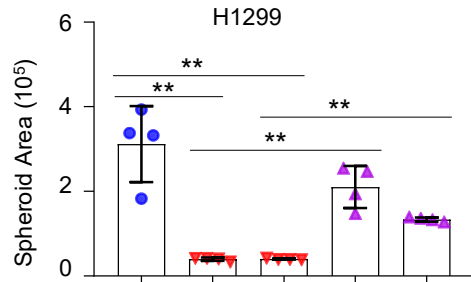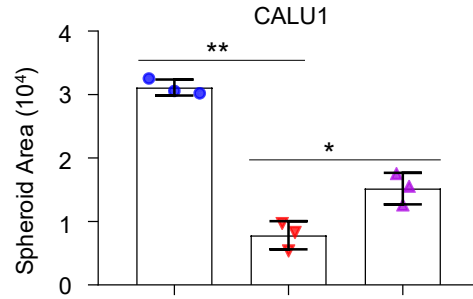

|          |   |   |   |   |   |
|----------|---|---|---|---|---|
| ASO Ctrl | + | - | - | - | - |
| ASO#1    | - | + | - | + | - |
| ASO#2    | - | - | + | - | + |
| Ev       | + | + | + | - | - |
| MYC      | - | - | - | + | + |

|          |   |   |   |
|----------|---|---|---|
| ASO Ctrl | + | - | - |
| ASO#1    | - | + | + |
| Ev       | - | + | - |
| MYC      | - | - | + |

**B**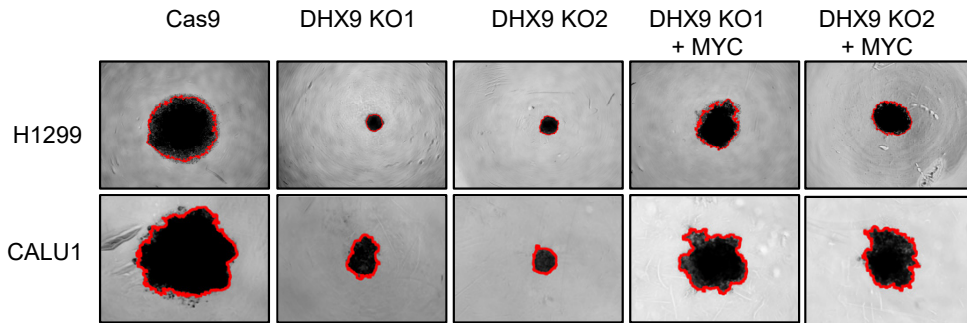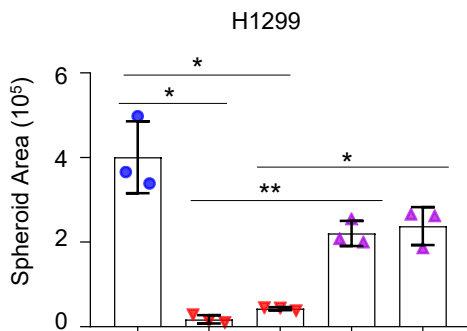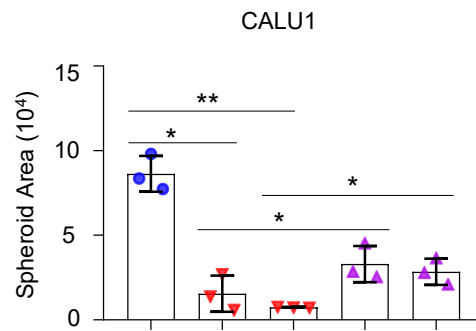

|          |   |   |   |   |   |
|----------|---|---|---|---|---|
| Cas9     | + | - | - | - | - |
| DHX9 KO1 | - | + | - | + | - |
| DHX9 KO2 | - | - | + | - | + |
| Ev       | + | + | + | - | - |
| MYC      | - | - | - | + | + |

|          |   |   |   |   |   |
|----------|---|---|---|---|---|
| Cas9     | + | - | - | - | - |
| DHX9 KO1 | - | + | - | + | - |
| DHX9 KO2 | - | - | + | - | + |
| Ev       | + | + | + | - | - |
| MYC      | - | - | - | + | + |

Supplement: Supplementary file 12 — Supplementary Figure 12 [file 41418_2023_1160_MOESM12_ESM.pdf]

**A**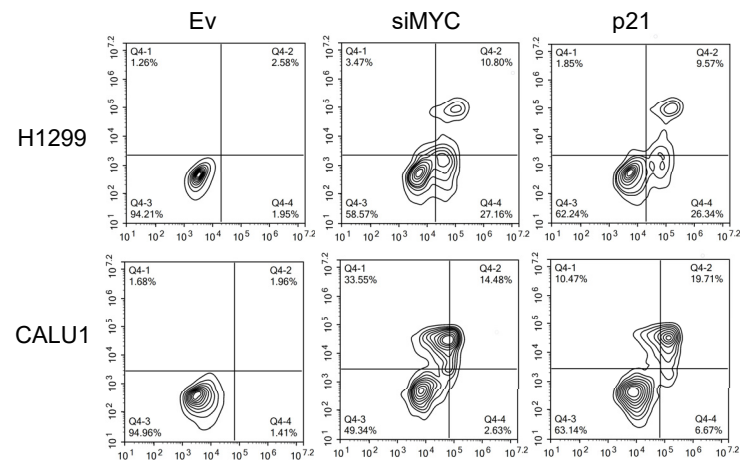**B**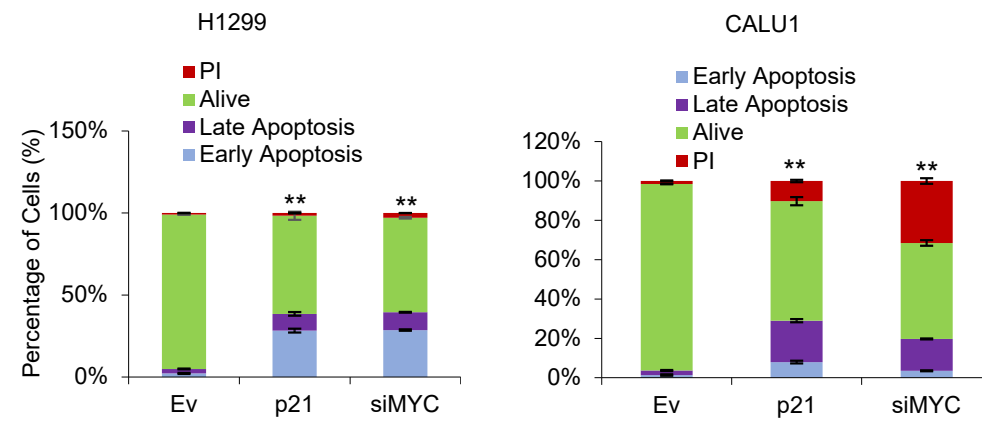**C**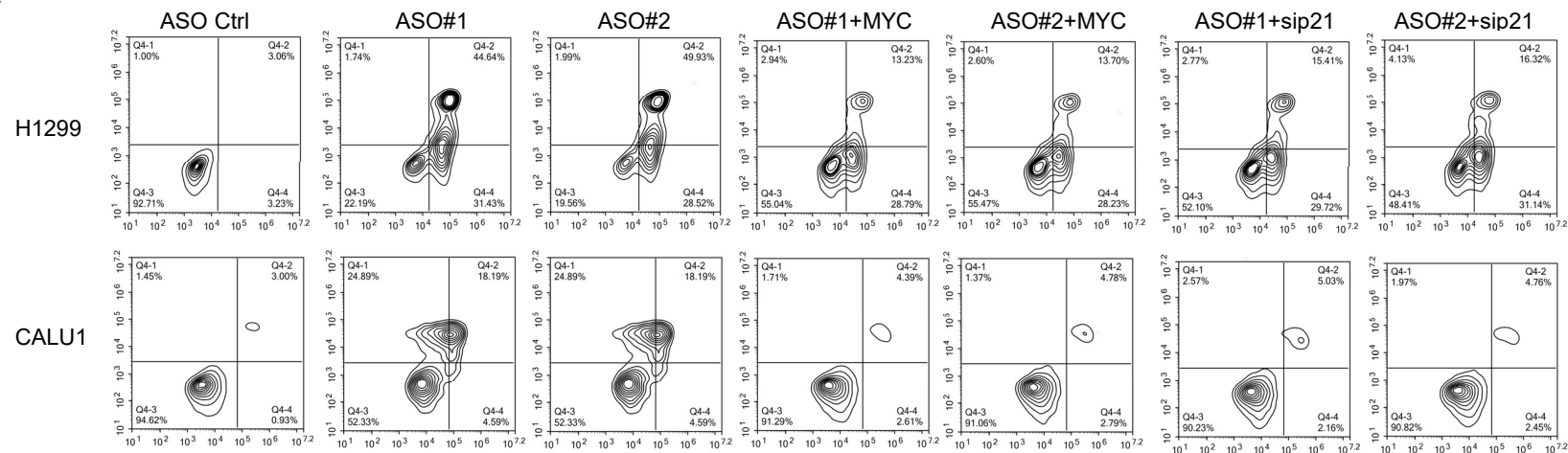**D**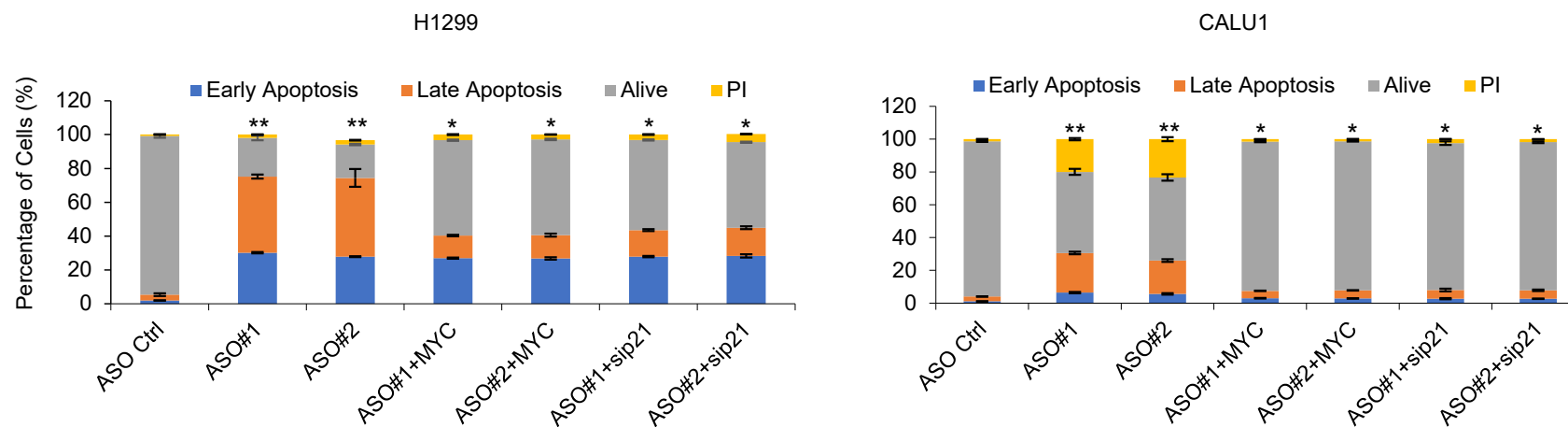

Supplement: Supplementary file 13 — Supplementary Figure 13 [file 41418_2023_1160_MOESM13_ESM.pdf]

**A**

Relative Expression

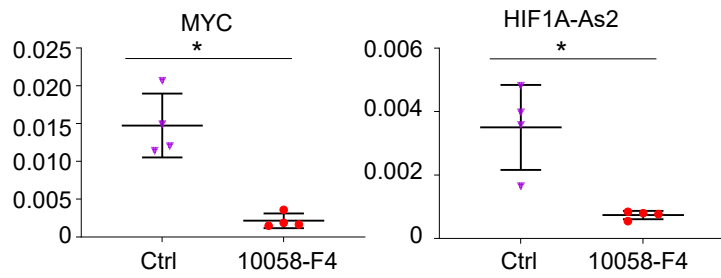**B**

Relative Expression

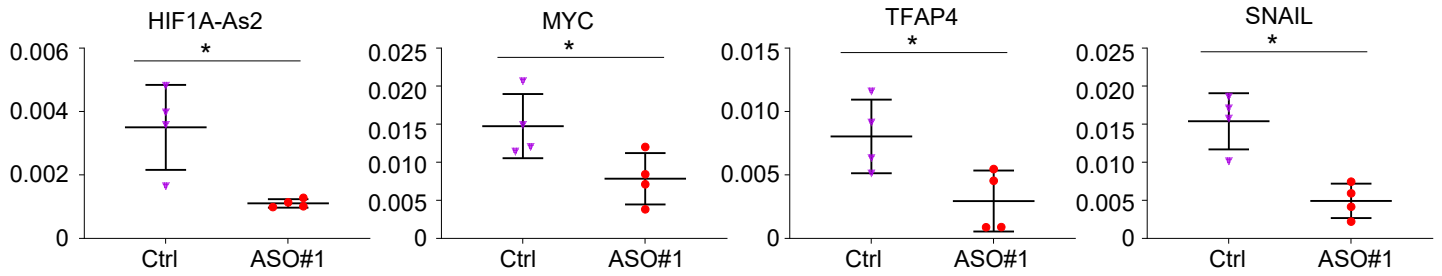**C**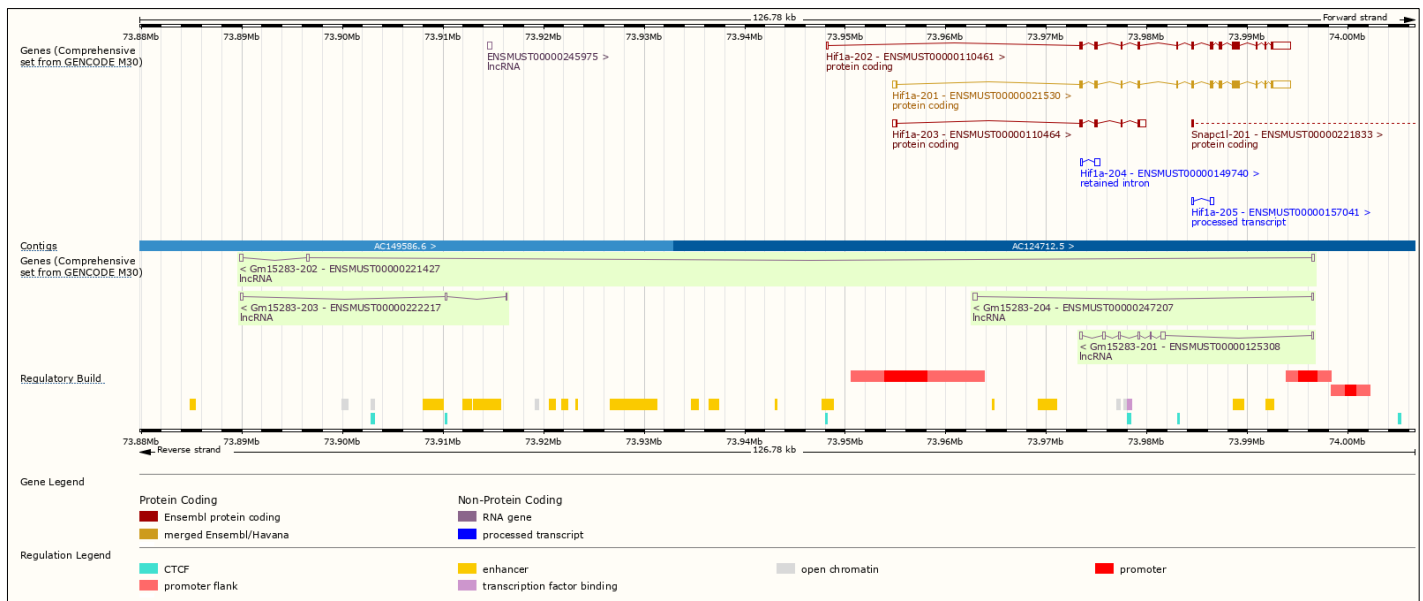**D**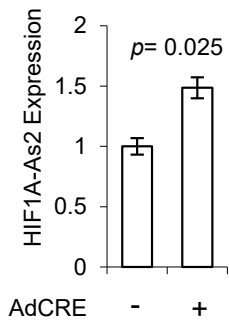**E**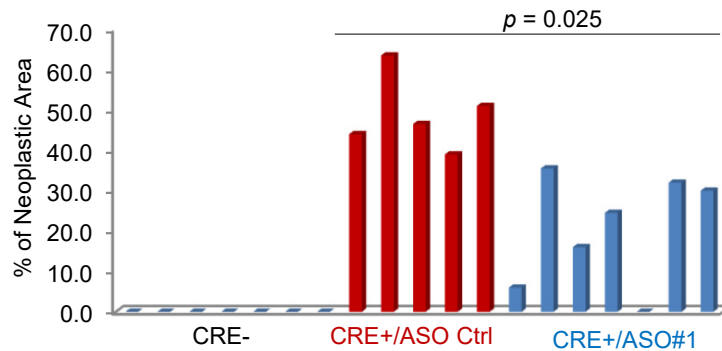**F**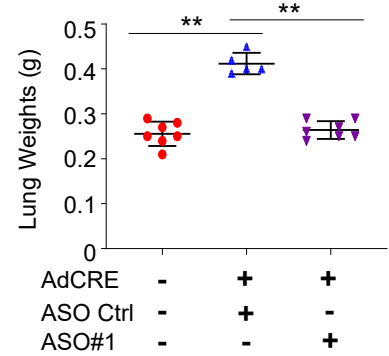**G**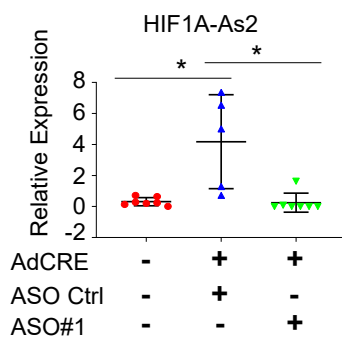**H**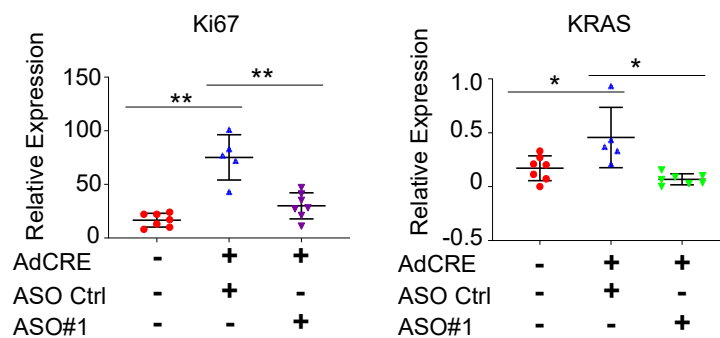

Supplement: Supplementary file 14 — Supplementary Figure 14 [file 41418_2023_1160_MOESM14_ESM.pdf]
