# Supplementary material for: Long non-coding RNA HIF1A-As2 and MYC form a double-positive feedback loop to promote cell proliferation and metastasis in KRAS-driven non-small cell lung cancer
Source: Cell Death Differ. 2023 Apr 11;30(6):1533–49. doi: 10.1038/s41418-023-01160-x (PMC10089381; doi:10.1038/s41418-023-01160-x)
Supplement: Supplementary file 6 — Supplementary Figure 6 [file 41418_2023_1160_MOESM6_ESM.pdf]

A

Group	Mice Number	Tumour Number	Tumour Average Size (mm^3)	Period (day)
H1299 Ev	7	2	184.89	40
H1299 HIF1A-As2	7	6	553.4783	40
H460 Ev	8	2	332.176	19
H460 HIF1A-As2	8	7	1461.46	40

B

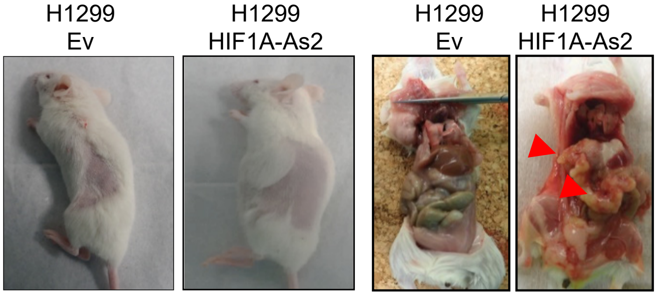

C

Group	Tumour	Mice Number	Ratio
H1299 Ev	Lung tumor	2	2/7
	Liver tumor	1	1/7
	Colon tumor	0	0/7
	Malignant Ascites	1	1/7
H1299 HIF1A-As2	Lung tumor	5	5/7
	Liver tumor	7	7/7
	Colon tumor	3	3/7
	Malignant Ascites	6	6/7
