# Supplementary material for: Long non-coding RNA HIF1A-As2 and MYC form a double-positive feedback loop to promote cell proliferation and metastasis in KRAS-driven non-small cell lung cancer
Source: Cell Death Differ. 2023 Apr 11;30(6):1533–49. doi: 10.1038/s41418-023-01160-x (PMC10089381; doi:10.1038/s41418-023-01160-x)

Figure 4C

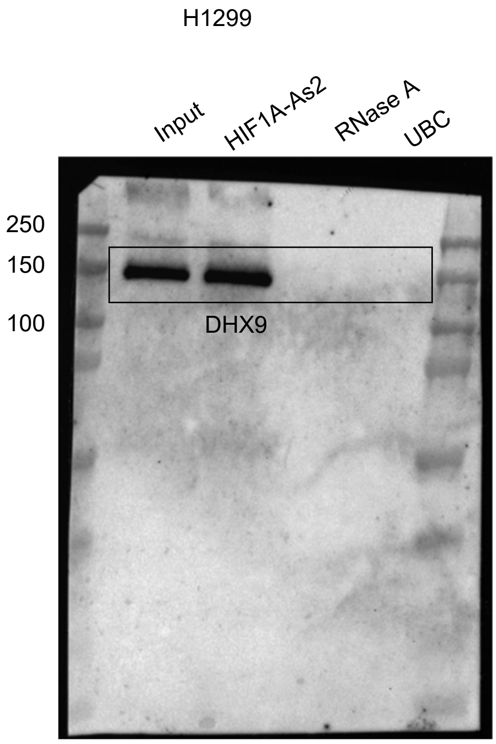

Figure 4E

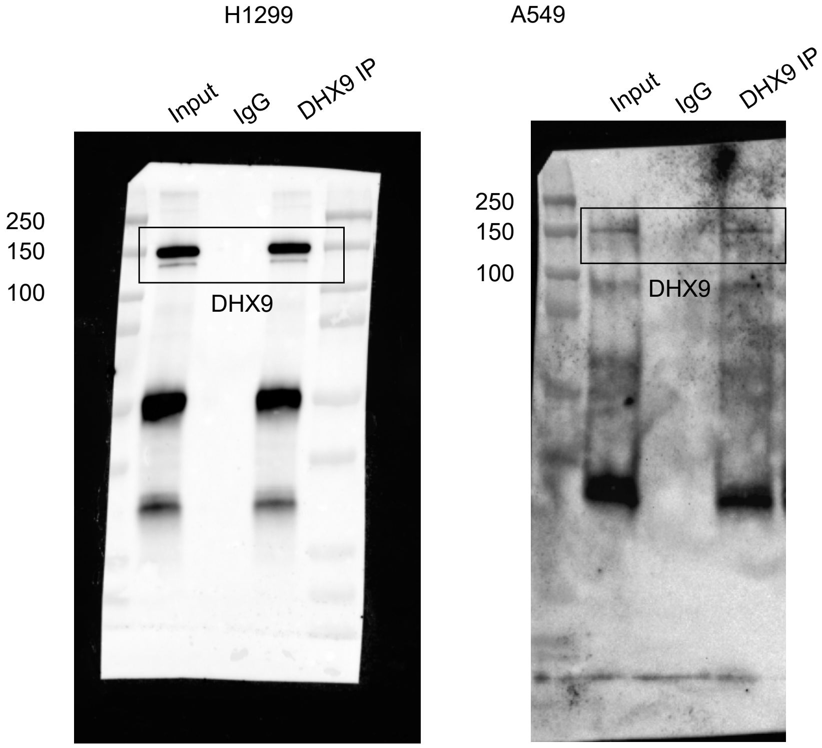

Figure 4H

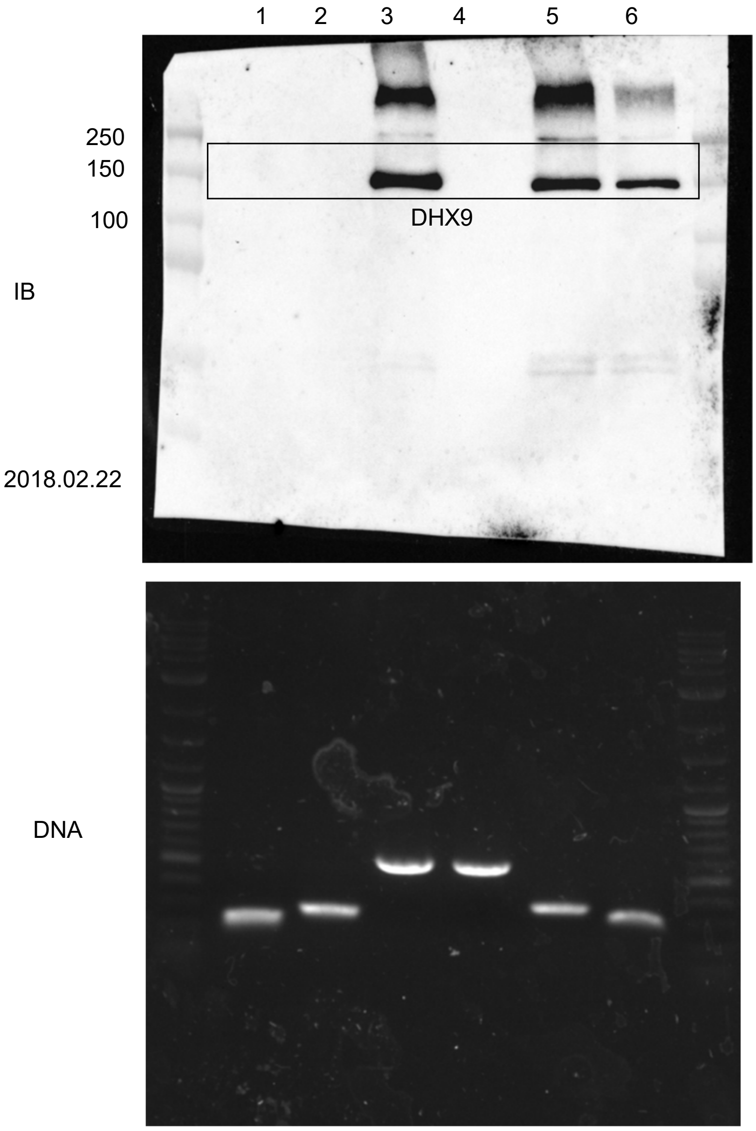

Figure 4I

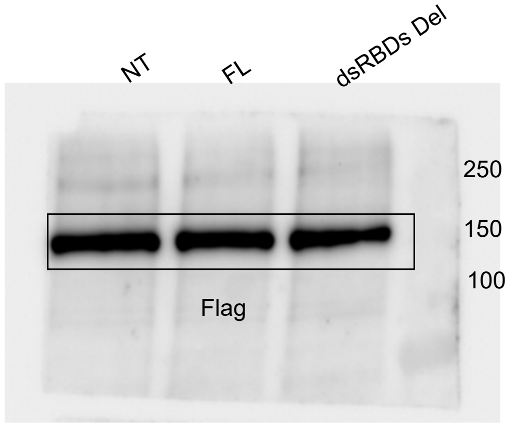

Figure 5B

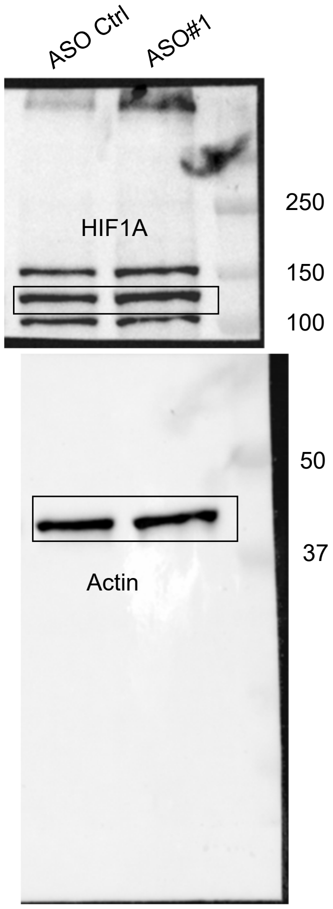

Figure 5J

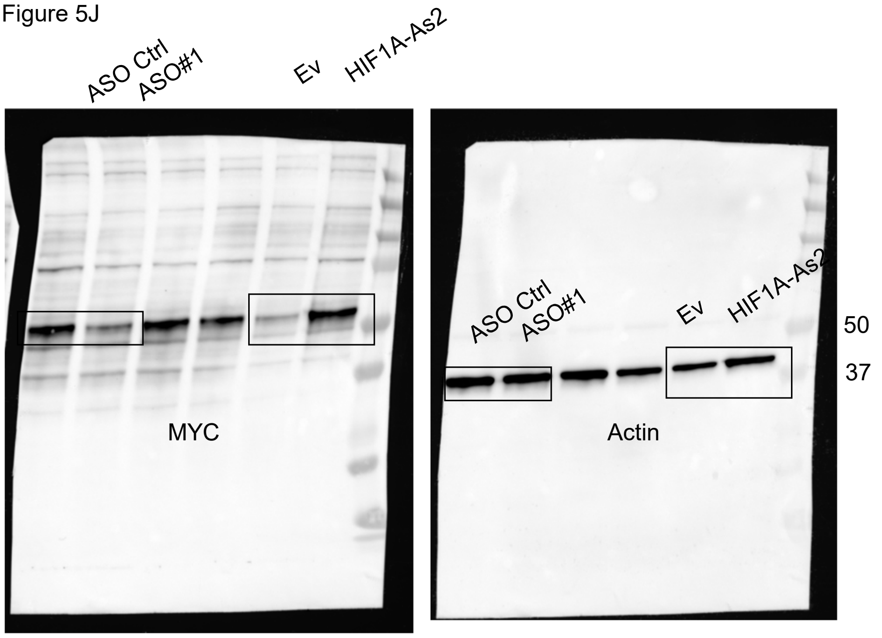

Figure 5K

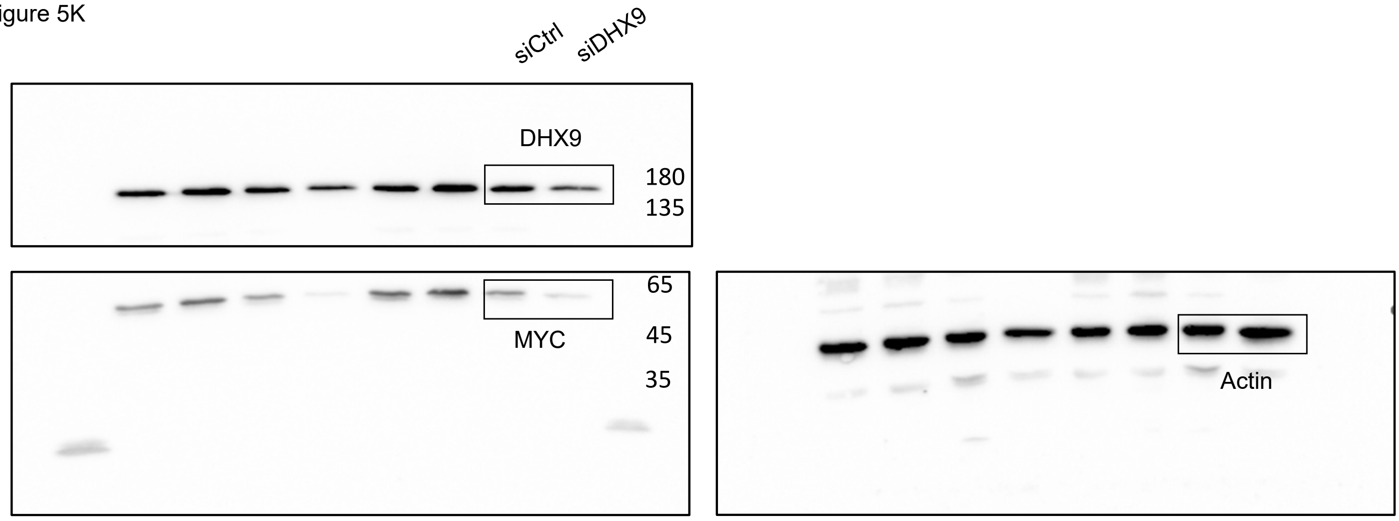

Figure 6B H1299

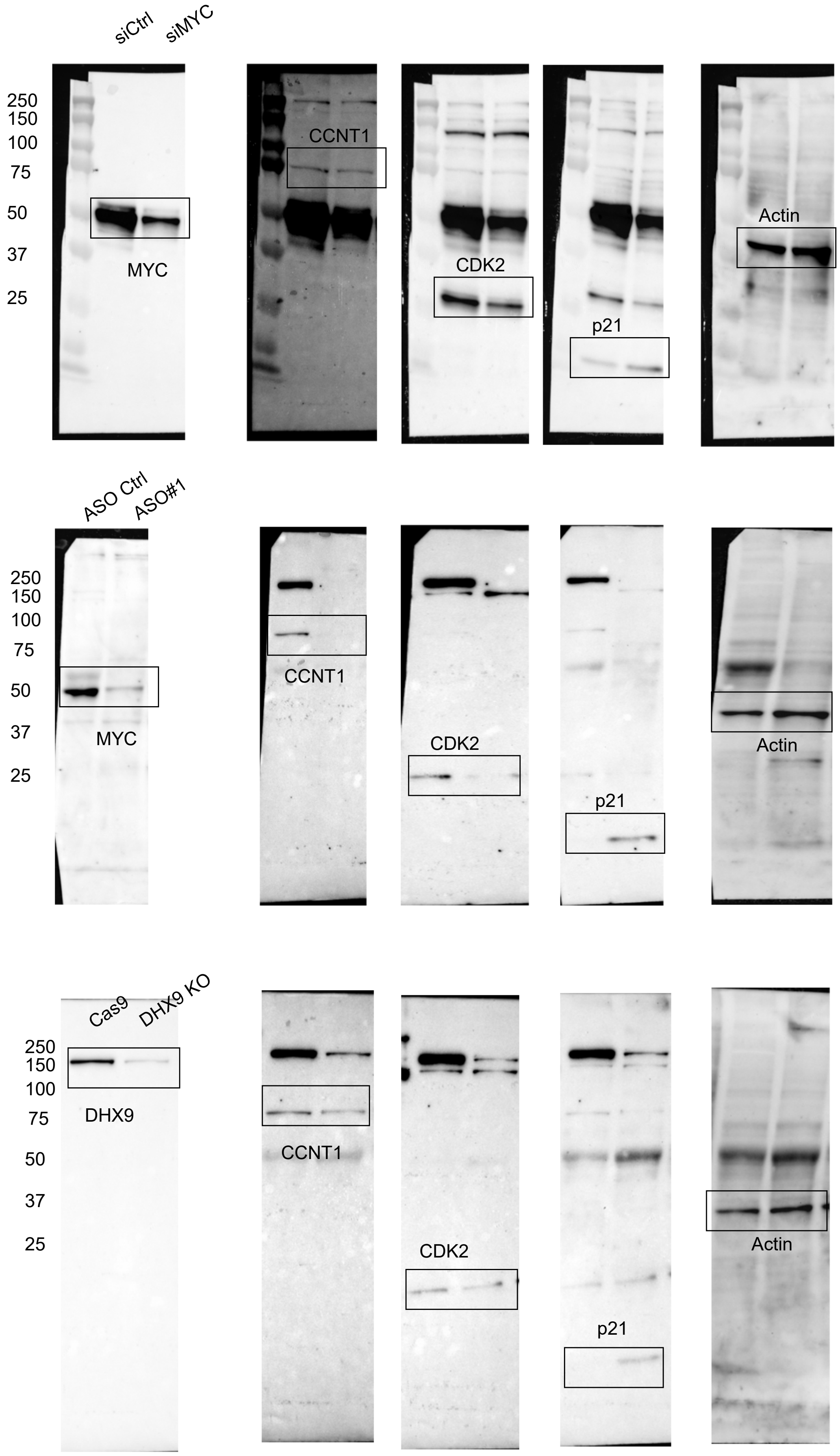

Figure 6B CALU1

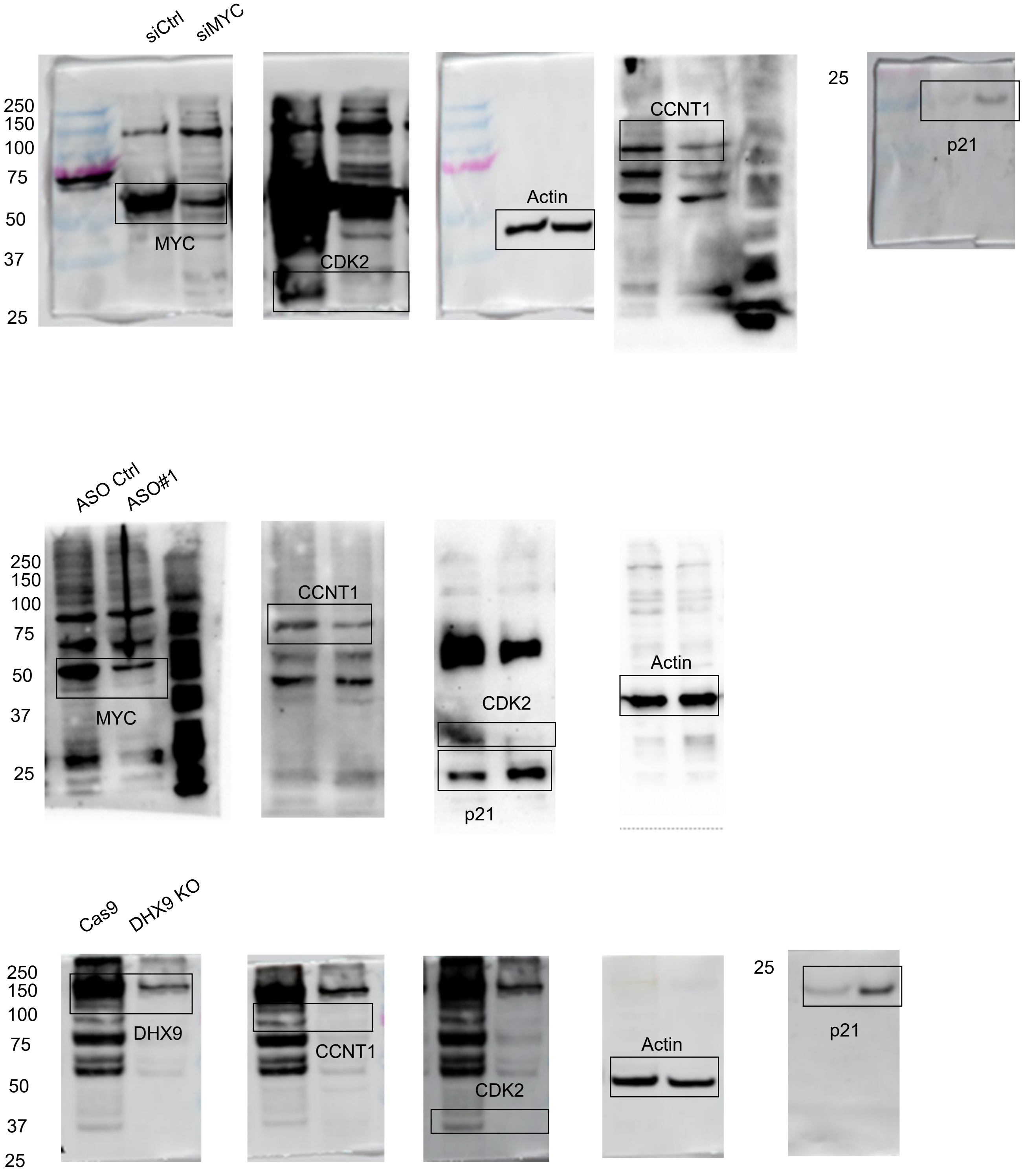

Figure 7B

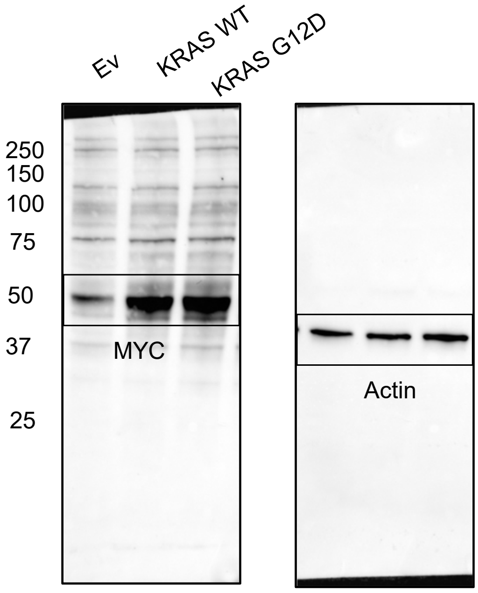

Ssupplementary Figure 5J

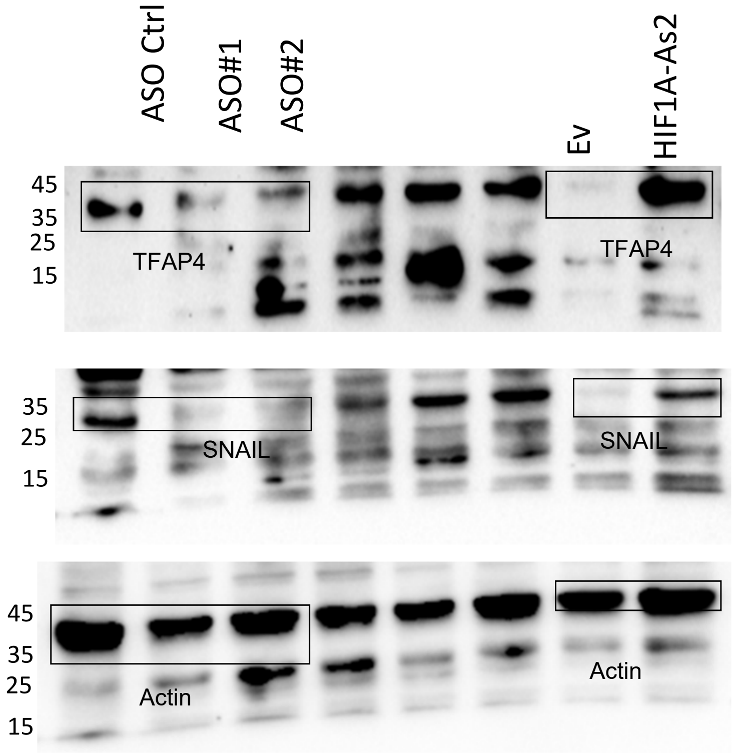

Supplement: Supplementary file 15 — Supplementary Figure 15 [file 41418_2023_1160_MOESM15_ESM.pdf]
