# Supplementary material for: Long non-coding RNA HIF1A-As2 and MYC form a double-positive feedback loop to promote cell proliferation and metastasis in KRAS-driven non-small cell lung cancer
Source: Cell Death Differ. 2023 Apr 11;30(6):1533–49. doi: 10.1038/s41418-023-01160-x (PMC10089381; doi:10.1038/s41418-023-01160-x)
Supplement: Supplementary file 17 — Supplementary Figure Legend [file 41418_2023_1160_MOESM17_ESM.docx]

**Supplementary Figure 1. *HIF1A-As2* is highly expressed in lung cancer.** A, Visualization of the signal of *HIF1A-As2* from KRAS WT and G12D RNA-seq through UCSC Genome Browser. Red box indicates the differently signal levels at *HIF1A-As2* exon 1. B, *In silico* analysis of the coding probability of *HIF1A-As2* transcript. C, *HIF1A-As2* is not conserved in chimp, mouse, fish and fly species. D, *HIF1A-As2* full length was determined by 5’ and 3’ rapid-amplification of cDNA ends (RACE) assay. *HIF1A-As2* total length is 533 nucleotides. Data are representative of 2 independent experiments. FL, full length. E, Cytoplasmic (Cyto) and nuclear (Nuc) cell fractionation followed RT-qPCR showing *HIF1A-As2* is predominately localised in the nucleus in multiple NSCLC cell lines. *Actin* and *MALAT1* were used as controls. F, RT-qPCR showing *HIF1A-As2* level after silencing of EGFR. G, Level of *HIF1A-As2* in H1299 and A549 treated with trametinib (20 μm) for 48 hours. H, KRAS expression in the TCGA LUAD (n=540) and LUSC (n=501) dataset compared to GTex Lung cohorts (n=427). I, Positive correlation between KRAS and *HIF1A-As2* in TCGA LUSC dataset (LUSC+GTex n=928). J, Representative images of H&E staining and *HIF1A-As2* smFISH in different stage of Biomax LUAD TMA samples (Biomax LUAD normal n=75, T1 n=20, T2 n=40, T3 n=12, T4 n=3). E-G, Data show mean ± S.D (n=3). **p value < 0.001, *p value < 0.05 by two-tailed Student’s t-test.

**Supplementary Figure 2. *HIF1A-As2* is required in cell proliferation in NSCLC.** A, GSEA analysis showing the enrichment of the G2M checkpoint signature in KRAS WT and KRAS G12D gene sets. B, Left, RT-qPCR of *HIF1A-As2* level in NSCLC cell lines; Right, Table indicating the status of *KRAS* mutation in NSCLC cell lines. C, smFISH representative images of *HIF1A-As2* upon transfection with *HIF1A-As2* ASO#1. DAPI, blue; *HIF1A-As2*, red. Scale bar, 75 μm. D, MTS cell proliferation assay after silencing of *HIF1A-As2* in BEAS2B and HEL299 cells. E, *HIF1A-As2* KD sensitize cells to Gefitinib and Cisplatin treatment in H1299 and CALU6 cells, respectively. F, *HIF1A-As2* KD by specific siRNA (Left) inhibited cell growth and promoted sensitivity to Cisplatin in H1299 cells (Right). G, Cell cycle analysis of H1299 cells upon silencing of *HIF1A-As2*. Data show mean ± S.D (B, F, G n=3; D n=6; E Left and Middle n=4, Right n=3). **p value < 0.001, *p value < 0.05 by two-tailed Student’s t-test.

**Supplementary Figure 3. Representative Annexin-V plots indicating apoptosis distribution in multiple cells upon *HIF1A-As2* knock down.**

**Supplementary Figure 4. *HIF1A-As2* promotes cell proliferation.** A, RT-qPCR analysis of *HIF1A-As2* level in H1299 and H460 cells stably expressing *HIF1A-As2*. B, Colony formation assay and corresponding quantification in H1299 and H460 cells that stably expressing *HIF1A-As2*. C, Restored *HIF1A-As2* recused the repressed colony formation by *HIF1A-As2* KD. Data show mean ± S.D (n=3). **p value < 0.001, *p value < 0.05 by two-tailed Student’s t-test.

**Supplementary Figure 5. *HIF1A-As2* regulates EMT by sponging microRNA-200c.** A, Bioinformatics prediction of *HIF1A-As2* targets using two different algorithms. B, Location and possible base pairing within a predicted miR-200c binding sequences in *HIF1A-As2*. C, Dual-luciferase reporter analysis showing miR-200c directly targets *HIF1A-As2*. D, miR-200c is enriched in *HIF1A-As2* pulldown lysates. Samples pre-treated with RNase A or pulldown with housekeeping gene *UBC* were used as negative controls. E, RT-qPCR analysis of *HIF1A-As2* level by ectopic miR-200c. *ZEB1* was used as a control. F, GSEA analysis showing the enrichment of the EMT signature in *HIF1A-As2* KD gene set. G and H, Representative pictures of Wound healing assay (G) and corresponding quantifications (H) 48 hours after silencing *HIF1A-As2* in H1299 and A549 cells. Wound closure was determined 24 hours after scratching. Mitomycin C was applied for 2 hours before scratching. I, Determination of migration and invasion of H1299 *HIF1A-As2* KD cells in a Transwell Boyden chamber assay. To analyze invasion, membranes were coated with Matrigel (3.3 ng/ml in respective medium). J, RT-qPCR (Top) and Immunoblotting (Bottom) analysis of mesenchymal marker TFAP4 and SNAIL upon *HIF1A-As2* KD in H1299 cells and *HIF1A-As2* overexpression in H460 and HBEC cells, respectively. K, Representative images depicting a morphological change in *HIF1A-As2* stably expressing cells. Data show mean ± S.D (n=3). **p value < 0.001, *p value < 0.05 by two-tailed Student’s t-test.

**Supplementary Figure 6. *HIF1A-As2* promotes tumor growths and metastasis.** A, Table showing *HIF1A-As2* promotes cell growth *in vivo*. B，Representative images of mice orthotopically injected with H1299-*HIF1A-As2* cells showing malignant ascites (Left) and distant metastases (Right) compared to control mice. C, Table reporting the distant metastases and relevant clinical symptoms of mice orthotopically injected with H1299-*HIF1A-As2* or control cells.

**Supplementary Figure 7. The oncogenic role of DHX9 in NSCLC.** A, RT-qPCR showing *HIF1A-As2* did not alter DHX9. B, RNA stability assay showing that DHX9 enhance *HIF1A-As2* RNA stability in cells incubation with 5 μg/ml actinomycin D for the indicated groups. Act D, actinomycin D. C, Positive correlation between *HIF1A-As2* and DHX9 in Biomax LUAD TMAs (n=150). D, Survival curve indicating patients with high expression of *HIF1A-As2* and DHX9 associated with a shorter lifespan. E. Colony formation assay of H1299 cells after transfection of *HIF1A-As2* full-length (FL) or the truncated construct *HIF1A-As2* Δ2. *HIF1A-As2* Δ2 refers to construct 2 in Figure 4H which lacking 271 nt from 3’end for DHX9 binding. F, DHX9 KO halted the induced cell proliferation by ectopic *HIF1A-As2*. G, Overexpression of DHX9 but not DHX9 dsRBDs del restored the inhibition of cell proliferation by DHX9 KO in H1299. H, Representative images showing lung tumors of the mice from the indicated groups. Dashed circle displays the tumor. I, Representative images of lungs and livers presenting distant metastases in the mice injected with H1299 Cas9 or DHX9 KO cells. J, Representative H&E staining of lungs from NSG mice after orthotopic injection of H1299 Cas9 or DHX9 KO cells. H-J, n=8 per group. Data show mean ± S.D in A, B, E, F and G (n=3). **p value < 0.001, *p value < 0.05 by two-tailed Student’s t-test.

**Supplementary Figure 8. *HIF1A-As2* regulates genes in trans.** A, Chart plots of gene signatures regulated by *HIF1A-As2* with GSEA analysis. B, RT-qPCR showing mRNA levels of the indicated TFs upon two *HIF1A-As2* ASOs transfection in H1299 cells. C, GSEA analysis showing the enrichment of the MYC signature in *HIF1A-As2* KD gene sets. D, Chart plot of the MYC signature by GSEA analysis in *HIF1A-As2* KD gene sets. E, Venny plots indicating overlapping between MYC targets and *HIF1A-As2* regulated genes (Left) or MYC targets and DHX9 regulated genes (Right). MYC targets were downloaded from the Harmonizome dataset, a collection of processed datasets about genes and proteins. F, Venny plots indicating overlapping between *HIF1A-As2* regulated genes and DHX9 regulated genes. G，ChIP assay indicating *HIF1A-As2* interacts with H3K4me3 and H3K27ac in H1299 cells. H, ChIP-qPCR analysis showing the enrichment of DHX9 on MYC promoter (-1800 to -1500 nt) upon ectopic *HIF1A-As2*. Data show mean ± S.D (n=3) in G and H. **p value < 0.001, *p value < 0.05 by two-tailed Student’s t-test.

**Supplementary Figure 9. MYC directly binds to the targets.** A, UCSC Genome Browser showing the ChIP-seq signals of MYC on the promoter regions of target genes. B, ChIP-qPCR showing the MYC binds to the promoter regions of the indicated genes. Data show mean ± S.D (n=3). **p value < 0.001, *p value < 0.05 by two-tailed Student’s t-test.

**Figure 10. *HIF1A-As2* and DHX9 modulate MYC and p21 *in vivo*.** A and B, Representative images of IHC staining with MYC and p21 antibodies from mice orthotopically injected with *HIF1A-As2* or control cells (A, H1299 cells, n=7 per group; B, H460 cells, n=8 per group). C, Representative IHC images of MYC and p21 from the mice orthotopically injected with H1299 Cas9 or DHX9 KO cells. n=8 per group. Data show mean ± S.D. **p value < 0.001, *p value < 0.05 by two-tailed Student’s t-test.

**Supplementary Figure 11. *HIF1A-As2* regulates cell proliferation through MYC.** A, MYC KD by siRNA or a specific inhibitor, 10058-F4 (10 μM, 48 hours) increased the luciferase activities of p21 reporter, whereas MYC overexpression reversed this inhibition. B, The repressed luciferase activities of p21 reporter by *HIF1A-As2* were rescued upon MYC KD. C-E, Colony formation experiments showing that *HIF1A-As2* promotes cell growth via MYC in CALU1 (C), H1299 (D) and H460 (E). Data show mean ± S.D (n=3). ***p* value < 0.001, **p* value < 0.05 by two-tailed Student’s *t-*test.

**Supplementary Figure 12. *HIF1A-As2* (A) and DHX9 (B) promote 3D spheroid formation through MYC in H1299 and CALU1 cells.** Data show mean ± S.D (n=3). ***p* value < 0.001, **p* value < 0.05 by two-tailed Student’s *t-*test.

**Supplementary Figure 13. *HIF1A-As2* regulates cell apoptosis via MYC and p21.** A and B, Representative Annexin-V plots (A) and quantification (B) upon MYC KD or p21 OE. C and D, *HIF1A-As2* regulated apoptosis through MYC and p21 in H1299 and CALU1 cells. Data show mean ± S.D (n=3). **p value < 0.001, *p value < 0.05 by two-tailed Student’s t-test.

**Supplementary Figure 14. *HIF1A-As2* ASO inhibits KRAS-driven tumorigenesis *in vivo*.** A, qPCR showing the gene level from the indicated group. B, *HIF1A-As2*, *MYC*, *TFAP4* and *SNAIL* level from the mice with *HIF1A-As2* ASO. C, Ensembl showing the aliment information of Gm15283 transcript, the corresponding gene of human *HIF1A-As2* in mouse genome. D, RT-qPCR showing *HIF1A-As2* expression in the lungs of KRAS^LSLG12D^ mice 8 weeks after intranasal administration of AdenoCRE. E, Bar plot depicting the percentage of neoplastic area in the indicated groups of mice. F, Graph showing lung weights (g) of the indicated groups of mice. G, RT-qPCR showing *HIF1A-As2* expression level in the plasma of the indicated groups of mice. H, Quantification of Ki67 immunostaining of lung sections from KRAS^LSLG12D^ mice (Left). KRAS relative expression in the indicated group by RT-qPCR (Right). A and B, n=4; E-H, Group GRE- n=7, Group CRE+/ASO Ctrl n=5, Group CRE+/ASO#1 n=7. Data show mean ± S.D. **p value < 0.001, *p value < 0.05 by two-tailed Student’s t-test.

**Supplementary Figure 15. Western Blot source data.**
